# Lyophilized Iron Oxide Nanoparticles Encapsulated in Amphotericin B: A Novel Targeted Nano Drug Delivery System for the Treatment of Systemic Fungal Infections

**DOI:** 10.3390/pharmaceutics12030247

**Published:** 2020-03-10

**Authors:** Pavan Balabathula, Sarah Garland Whaley, Dileep R. Janagam, Nivesh K. Mittal, Bivash Mandal, Laura A. Thoma, P. David Rogers, George C. Wood

**Affiliations:** 1 Plough Center for Sterile Drug Delivery Systems, University of Tennessee Health Science Center, Memphis, TN 38163, USA; dileep.janagam@gmail.com (D.R.J.); nmittal@uthsc.edu (N.K.M.); bm2306@gmail.com (B.M.); 2 Department of Pharmaceutical Sciences, University of Tennessee Health Science Center, Memphis, TN 38163, USA; swhaley5@uthsc.edu (S.G.W.); lthoma@uthsc.edu (L.A.T.); drogers@uthsc.edu (P.D.R.); gwood@uthsc.edu (G.C.W.)

**Keywords:** nanoparticle(s), theranostics, targeted drug delivery, antiinfective(s), lyophilization, albumin, drug delivery system(s), drug transport, colloid(s), coating

## Abstract

We formulated and tested a targeted nanodrug delivery system to help treat life-threatening invasive fungal infections, such as cryptococcal meningitis. Various designs of iron oxide nanoparticles (IONP) (34–40 nm) coated with bovine serum albumin and coated and targeted with amphotericin B (AMB-IONP), were formulated by applying a layer-by-layer approach. The nanoparticles were monodispersed and spherical in shape, and the lead formulation was found to be in an optimum range for nanomedicine with size (≤36 nm), zeta potential (−20 mV), and poly dispersity index (≤0.2), and the drug loading was 13.6 ± 6.9 µg of AMB/mg of IONP. The drug release profile indicated a burst release of up to 3 h, followed by a sustained drug release of up to 72 h. The lead showed a time-dependent cellular uptake in *C. albicans* and *C. glabrata* clinical isolates, and exhibited an improved efficacy (16–25-fold) over a marketed conventional AMB-deoxycholate product in susceptibility testing. Intracellular trafficking of AMB-IONP by TEM and confocal laser scanning microscopy confirmed the successful delivery of the AMB payload at and/or inside the fungal cells leading to potential therapeutic advantages over the AMB-deoxycholate product. A short-term stability study at 5 °C and 25 °C for up to two months showed that the lyophilized form was stable.

## 1. Introduction

The systemic and invasive fungal infections (IFIs) are associated with high morbidity and mortality, particularly in immunocompromised hosts who are at substantial risk for these infections. The mortality rates of some of these IFIs go above 60% in certain situations and represent one of the greatest challenges facing clinicians who care for immunocompromised patients [1]. Cancer chemotherapy patients, those with HIV infection, and acute leukemia or hematopoietic stem cell transplant receivers are at substantial risk for IFIs. The incidence of IFIs has been further increased with aggressive treatment strategies, together with improvements in medical care that extend the survival of critically ill patients, thus leading to increased numbers of immunocompromised patients [2]. Additionally, an increasing variety of fungal species has been found, particularly in these patients [3,4,5]. One of the most striking estimates recently was the global burden for cryptococcal meningitis in HIV-infected patients, with about one million new cases each year. It was estimated that 625,000 deaths occur among these one million cases every year [6].

To date, no ideal antifungal agent exists to cure IFIs [1]. Older agents are toxic and newer agents including azoles and echinocandins come with varied pharmacokinetic profiles, drug interactions, a narrow spectrum of activity, and limited routes of administration, constraining their application [1]. Amphotericin B (AMB) antibiotic was discovered in 1955 and it has been considered a gold standard for the treatment of IFIs since its FDA approval in 1959. It has the broadest spectrum of activity against fungi species including *Candida* species, *Cryptococcus neoformans*, and *Coccidioides immitis* [7]. The conventional formulation, amphotericin B-deoxycholate (AMB-D), is associated with a high incidence of infusion-related adverse effects and dose-related nephrotoxicity, thus limiting its usage. Other problems, such as pharmacokinetic variability, drug interactions, and limited routes of administrations, also limit its usage. To address these problems, lipid formulations (AmBisome^®^, Amphocil^®^, and Abelcet^®^) of AMB have been developed, but the relative efficacy and safety of these drugs have not been adequately studied [1,2].

Despite several advancements in antifungal therapy, the best therapy for IFIs still remains a challenge. The development of new antifungal drugs for the treatment of IFIs has not kept pace with the progress in fungal therapy, due to the nonspecific drug distribution resulting in low antifungal drug concentrations and systemic toxicity. Therefore, a formulation with improved therapeutic efficacy and reduced adverse effects is needed, preferably one that can be delivered by developing a targeted nanoparticle delivery system to the specific site of action, lowering the required dose and keeping AMB away from healthy cells. Additionally, it would be advantageous to integrate an imaging property within the delivery system to follow the path of nanoparticles upon administration, and aid in diagnosing and monitoring the severity of the infection.

The advances in the treatment of IFIs, like fungal meningitis, are advancing both in terms of new agents against these infections and in innovative ways of delivering both old and new agents. In the recent development of nanomedicines, iron oxide nanoparticles (IONP) have gained increasing attention for biomedical applications [8,9]. These particulate systems are ideal for a higher accumulation in the target tissues or organs due to their host cell tropism, biophysical nature, and low toxicity [10]. IONP is among the few materials injected into the body that is easily incorporated into the body’s natural metabolic pathways [11].

The active pharmaceutical ingredients (APIs) bind to the surface of the IONP, which are then released at the target site with the application of external localized magnetic field gradients or through specific targeting. This approach offers the possibility for a reduction in nonspecific toxicities by administering lower but more accurately targeted doses [9,10]. Additionally, IONP improves the magnetic contrast for MRI and will potentially be useful as a diagnostic/imaging agent as well. The National Institutes of Health has placed emphasis on developing nanoparticle medicines that can also be imaged for diagnosis and monitoring.

As a proof of concept, we developed a novel Amphotericin B encapsulated IONP-based nanoformulation, attempted the treatment of some of the deadliest IFIs, and tested on clinical isolates of *Candida* [12]. Due to the inherent nature of the IONPs, they have the potential to be used as diagnostic aids. This study also demonstrated that established antibiotics can be converted into nanomedicines that will be more effective in killing drug-resistant bacteria. This research has a high priority for the Centers for Disease Control, as many of our current antibiotic formulations do not eliminate certain deadly microbes [13].

The advantages of this system over existing line of therapy is that (i) it has the potential for enhanced cellular uptake due to the targeted-binding and nanozised nature; (ii) it represents a potential for use in diagnosing and monitoring the infection due to the magnetic contrast nature of IONPs; and (iii) it can be loaded with multiple drugs/different targeting agents that have different binding domains on the albumin. This article explored the initial formulation feasibility and antifungal efficacy of the nanosystem in vitro, and the diagnosis potential of the nanosystem was not evaluated.

IONP (35–50 nm) containing AMB were formulated and coated with BSA (bovine serum albumin, a model protein for human serum albumin), using a layer by layer approach. This was converted into a targeted drug delivery system by targeting with AMB, itself, in low quantity (AMB-IONP). The surface layer AMB acts as both an effective drug and as a targeting agent for the nanoparticles. AMB is a naturally occurring surfactant and has a strong affinity for ergosterol in the fungal membrane. This allows the particles to migrate to the infection sites and stay there as they slowly dissolve. The AMB binding ability greatly simplifies the particle design in that an additional targeting layer does not need to be added to the nanoparticles. With this design, the layering process and eventual small-scale production can be simplified for animal studies and clinical trials. Therefore, it is expected that these processes and properties can be more amenable to production scale-up, as larger batches will be needed for further product testing. The formulations here were evaluated for in vitro cell uptake, efficacy, mechanism, and intracellular trafficking in fungal clinical isolates. The formulations were lyophilized to develop a stable AMB-loaded targeted IONP (AMB-IONP). We anticipated that these in vitro studies would produce a proof of concept to allow preclinical animal studies. This nanosystem has the potential to be developed into a theranostic product that could cure IFIs with better therapeutic efficacy and lesser toxicity when compared to the current therapies.

## 2. Materials and Methods

### 2.1. Materials

The oleic acid coated IONP, activation buffer (50 mM sodium borate buffer, pH 5.5), coupling buffer (50 mM sodium borate buffer, pH 8.5), quenching buffer (1 M 2-(2-aminoethoxy) ethanol, pH 9.5), wash/storage buffer (borate buffer, pH 7.4), and SuperMag Separator™ magnetic separator were obtained from Ocean NanoTech (Springdale, AR, USA). EDAC (1-ethyl-3-(3-dimethylaminopropyl) carbodiimide) was purchased from Acros (Pittsburg, PA, USA) and sulfo-NHS (N-hydroxysulfosuccinimide) was purchased from Thermo Scientific (Rockford, IL, USA). BSA (Cohn fraction V, lyophilized powder, MW ~ 66,000, purity 95–99%), glutaraldehyde solution (25% in water), tris-acetate- Ethylenediaminetetraacetic acid (TAE), dimethyl sulfoxide (DMSO), Coumarin 6 dye, RPMI (Roswell Park Memorial Institute) 1640, amiloride, chlorpromazine, genistein, methyl-β-cyclodextrin (MβCD), nocodazole, and sucrose were purchased from Sigma-Aldrich (St. Louis, MO, USA). AMB United States Pharmacopeia (USP) analytical standard was obtained from MP Biomedicals (Santa Ana, CA, USA) and high-performance liquid chromatography (HPLC) grade methanol, acetonitrile, water, DMSO, acetic acid, phosphate buffer saline (PBS), and 2 mL tubular vials were obtained from Fisher (Pittsburg, PA, USA). Molecular biology grade agarose, standard, low electroendosmosis was purchased from VWR International (Randor, PA, USA).

Fetal bovine serum (FBS) and the fungal clinical isolates *Candida albicans* (SC5314) and *Candida glabrata* (66032) were obtained from American Type Culture Collection (Manassas, VA, USA). *Candida krusei* (CK1), *Candida parapsilosis* (CP1), and *Candida tropicalis* (CT1) were shared by Dr. Daniel J. Diekema at the University of Iowa (Iowa City, IA, USA). The AMB-D was purchased from X-GEN Pharmaceuticals, Inc. (Horseheads, NY, USA). LysoTracker^®^ Deep Red was obtained from Thermo Fisher Scientific (Waltham, MA, USA). Vectashield^®^ cell mounting medium with 4’,6-diamidino-2-phenylindole (DAPI) was acquired from Vector Labs (Burlingame, CA, USA). T-Zero aluminum pans were bought from TA Instruments (New Castle, DE, USA). 13 mm lyophilization rubber stoppers and seals were obtained from the West Pharmacetucial Services, Inc (Cincinnati, OH, USA).

### 2.2. Formulation Design

**Figure 1:**
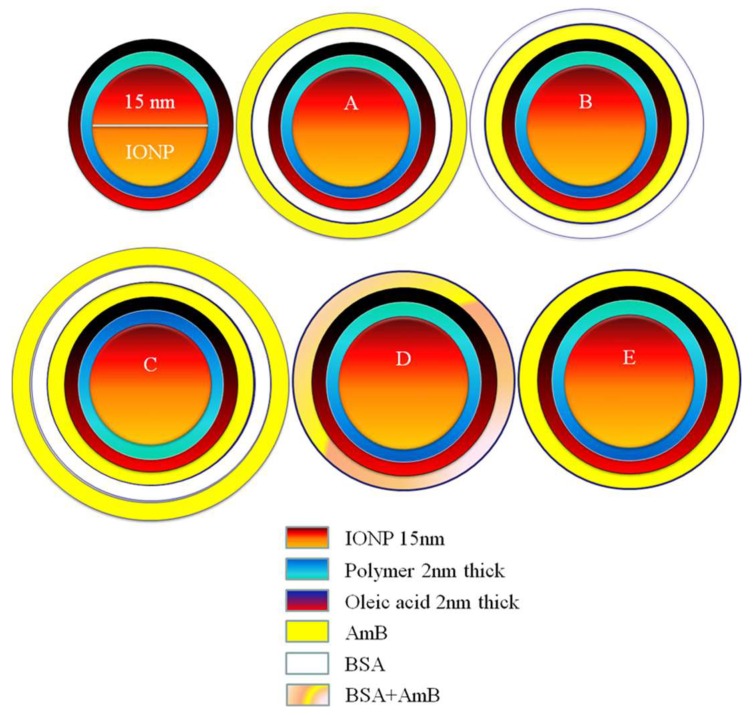
Formulation designs A, B, C, D, and E of iron oxide nanoparticles (IONP) coated and targeted with amphotericin B (AMB-IONP). IONP represents oleic acid-coated IONP with a core size of 15 nm.

The unique designs A, B, C, D, and E of AMB-IONP shown in Figure 1 were prepared using a layer-by-layer approach. The protein conjugation protocol used for the development of AMB-IONP formulation designs was adapted from Ocean NanoTech protocol [14]. Complete details were provided in the Supplementary (Supp.) sections for the procedure of making nanoformulations (Supp. Section S1), characterization of drug loading (Supp. Section S2), and particle size and Zeta potential (Supp. Section S3). The bovine serum albumin was selected as a model protein (low-priced alternative to human albumin) for conjugation of the drug.

### 2.3. Conjugation Efficiency

The success of the conjugation of BSA to IONP was monitored with agarose gel electrophoresis by running all the formulation designs alongside controls, IONP without the drug, and unlabeled IONP with BSA after 30 min of reaction. The agarose gel electrophoresis protocol was adapted from Ocean NanoTec [15]. Briefly, enough 1X Tris-Acetate was diluted from 10× for a 40 mL gel to completely fill the gel chamber and was set aside. 1–1.5% agarose gel was prepared by weighing out approximately 0.4–0.6 g of the agarose powder and then transferring the agarose into a 100 mL Erlenmeyer flask. We added 40 mL of 1X TAE buffer to the flask containing the agarose powder, swirled until homogenous, and heated to dissolve the agarose in the buffer. The gel chamber was filled with 1X TAE buffer about halfway and added the agarose buffer into the gel tray with the well comb fixed and allowed it to cool. The combs were removed after gel solidification. The tray was inserted properly into the gel chamber and enough 1X TAE buffer was poured into the chamber to cover the gel and fill the wells. The samples of 5 µL of IONP and formulation designs were pipetted into the wells of the gel. The gel was allowed to run at 100 V for 30 min.

### 2.4. Transmission Electron Microscopy (TEM)

The morphology of the IONP and design C and design D of AMB-IONP were characterized by TEM using a negative staining technique. A sequential two droplet method was used for staining the samples with uranyl acetate on a 400-mesh formvar support film on a copper specimen grid from Electron Microscopy Sciences (Hartfield, PA, USA), and then they were air dried. The TEM images were obtained at various magnifications using a JEOL 2000EX transmission electron microscope (Peabody, MA, USA) equipped with a high-resolution digital camera.

### 2.5. In Vitro Drug Release

Drug Release in 1X PBS: The drug release was studied in dissolution media containing 1X PBS at pH 7.4 using a previously reported dialysis method [16]. The in-house set up for the in vitro drug release experiment is shown in Supp. Figure S1. A 100 µL portion of the designs A, B, or C of AMB-IONP dispersion was pipetted into each designated Slide-A-LyzerTM MINI dialysis microtube with a molecular weight cutoff of 3500 Da (Pierce, Rockford, IL, USA). Mini-dialysis tubes were aligned in a floater which was placed in a beaker containing 1000 mL of the dissolution medium (PBS pH 7.4) above a magnetic stirrer stirring at 75 rpm. The whole set up was arranged inside an oven maintained at 37 °C. At a predefined time point, mini-dialysis tubes containing samples were taken out, then extracted in DMSO with sonication for 30 min at ambient temperature. After magnetic separation for 10–12 h at 4 °C, the samples were injected into an HPLC to quantify the amount of drug released at specified time points [17].

Drug Release in 1× PBS with 4% BSA: The lead formulation (design D) based on the cellular uptake was used. To further control the drug release, the BSA on design D was cross-linked with glutaraldehyde (0.47% of BSA). The addition of a chemical cross-linker such as glutaraldehyde provides better stability, shape, insolubility at elevated temperatures, and reduced swelling. The glutaraldehyde was added to the AMB-IONP dispersion and mixed for 12 h at ambient temperature. Then the particles were washed twice with wash/storage buffer and subjected to magnetic separation for 10–12 h at 4 °C. The liquid was aspirated out and the particles were dispersed in wash/storage buffer and stored at 4 °C until further use. Drug release was studied for samples both with and without a cross-linking agent in dissolution media containing 4% BSA in 1× PBS of pH 7.4, using the dialysis method described above. A 100 µL aliquot of the sample was pipetted into each designated Slide-A-LyzerTM MINI dialysis microtube and the same procedure as mentioned above with the PBS medium was followed.

### 2.6. Colloidal Stability in FBS

The colloidal stability of design D of AMB-IONP in serum was performed according to the previously reported method [18,19]. The AMB-IONP were suspended in 50% FBS with a final nanoparticle concentration of 1 mg/mL. The particles were concentrated to 2 mg/mL and an equal volume of FBS was then added. The samples were incubated at 37 °C in a shaker with light shaking. At predetermined time points, the samples were subjected to UV-Visible Spectrophotometer analysis. Due to the interference of high concentration of plasma proteins at 50% FBS, the absorbance measurements were conducted at 560 nm for each time point. The measurements were performed in triplicate at room temperature.

### 2.7. Preparation of Fluorescently Labeled AMB-IONP for Imaging

For imaging cellular uptake and intracellular localization in fungal clinical isolates, fluorescent-labeled AMB-IONP (design D) and placebo were prepared by mixing particles with Coumarin 6 dye dissolved in DMSO for 2 h. Coumarin-6 is a green fluorescent dye. The amount of dye used was 1% by weight of IONP. After adsorption of dye on the particles, the free dye was washed with wash/storage buffer and removed via the magnetic separation technique as previously described. The step was performed at least twice to ensure the removal of the free dye. Finally, the fluorescently labeled AMB-IONP were dispersed in wash/storage buffer and stored at 2–8 °C until further use.

### 2.8. Cellular Uptake of AMB-IONP by Confocal Laser Scanning Microscopy

Qualitative uptake and visualization of design D formulation of AMB-IONP were investigated by imaging using a confocal laser scanning microscopy method [20]. We placed 600 µL of *C. albicans* and *C. glabrata* clinical isolates at a cell density of 104/mL in each well for each time point. The cells were grown for another day to establish enough cell density for the study performed at 0.5- and 4-h time points. Both isolates were treated with the formulations at each time point. Fifteen minutes before the end of the time point, each well was treated with LysoTracker^®^ deep red (50 nM), a marker for secondary endosomes and lysosomes. Following the incubation period, the isolates were washed three times with 1X PBS and centrifuged at 13,000 rpm for 15 min. The pellet that formed at the end of the third wash was treated with one drop of Vectashield^®^ cell mounting medium with DAPI to visualize the nuclei as blue fluorescent shapes. The mixture was well mixed with a pipette and then placed on a microscope slide and covered with a coverslip. The slide was dried in a fume hood for 15–20 min. After drying, the sides of the coverslip were immobilized to the slide with the help of clear nail polish. The slides were again allowed to dry for another 15–20 min and stored in the freezer until further use. The slides were then observed with a Zeiss LSM 710 confocal laser scanning microscope (Carl Zeiss SMT Inc., Thornwood, NY, USA) by using a Plan-Apochromat 60x/1.4 oil DIC objective lens. The obtained images were processed with the help of Zeiss LSM Zen software in 2010.

### 2.9. Cellular Uptake of AMB-IONP by Flow Cytometry

For the quantitative evaluation of cellular uptake, a flow cytometry method was used [21]. Both isolates of *C. albicans* and *C. glabrata* were prepared and treated in a similar way as previously described for the confocal imaging studies. The uptake was studied at 0, 0.5, 1, 2, and 4 h. At the end of three washes with 1X PBS, the pellet was re-dispersed in 600 µL of 1X PBS. Each sample was then subjected to flow cytometry analysis to determine mean fluorescence intensity (MFI) with the help of BD Accuri^TM^ C6 flow cytometer (Becton Dickinson Inc., Franklin Lakes, NJ, USA) with FL1-A channel. Each sample was run in triplicate and the obtained data were processed with BD AccuriTM CFlow Plus analysis software.

### 2.10. In Vitro Efficacy of AMB-IONP in Clinical Isolates of Candida

Susceptibility testing control, AMB-D and all formulation designs (A through E) of AMB-IONP were evaluated for efficacy using a microbroth dilution assay according to the CLSI guidelines outlined in M27-A3 with a few modifications [22]. The isolates tested include *C. albicans* SC5314, *C. glabrata* 66032, *C. krusei* CK1, *C. parapsilosis* CP1, and *C. tropicalis* CT1. Additionally, *C. albicans* CA1008 and Calowa60, which contain less than 1% ergosterol, were also used for testing. The cultures were streaked on Sabouraud Dextrose plates and allowed to grow for 24 h. Colonies were then picked into sterile water and diluted to an optical density of 0.1 when measured at 600 nm. The cultures were diluted to a final inoculum of 0.5 × 10^3^–2.5 × 10^3^ cells/mL in RPMI 1640 with 2% glucose, MOPS (3-(*N*-morpholino) propanesulfonic acid), pH 7.0. Ninety-six well plates were incubated with increasing concentrations of the test compounds or controls at 35 °C for 48 h.

The absorbance at 600 nm was read with a microplate reader (Biotek, Winooski, VT, USA). The background absorbance due to the medium was subtracted from all readings. The minimum inhibitory concentration (MIC) was defined as the lowest concentration that prevented any discernible growth. A 90% inhibitory concentration (IC90) was calculated using SigmaPlot 12.0 at 24 h of treatment. Heat maps were prepared using Java TreeView. In addition, preliminary studies including transferrin as a targeting ligand and curcumin as a second drug load were also conducted with design D against clinical isolates of *C. albicans* SC5314, *C. glabrata* 66032, *C. krusei* CK1, *C. parapsilosis* CP1, and *C. tropicalis* CT1. After the preparation of AMB-IONP of design D, the particles were dispersed in 100 µL of transferrin solution (100 µg/mL) in water and incubated at ambient temperature for 2 h, followed with washing the unbound transferrin with similar washing steps described for other formulations.

To test whether multiple drugs can be loaded on to the formulation design D, curcumin was loaded along with AMB and subjected to susceptibility testing. AMB and curcumin have different binding domains on HSA and BSA, and AMB retains its antifungal activity [23]. In addition, curcumin can potentially reduce the toxic side effects as it will delay the lysis of red blood cells by AMB.

### 2.11. Cell Association Study of Fluorescently Labeled AMB-IONP

The uptake mechanism of design D particles of AMB-IONP into *C. albicans* and *C. glabrata* clinical isolates was determined by conducting receptor-mediated endocytosis cellular association studies at 4 °C and 35 °C [24,25]. The fluorescently labeled particles and cells for in vitro study were prepared as reported in Section 2.9. The study was performed in two separate cell culture plates maintained at 35 °C and pre-cooled to 4 °C, respectively, before the treatment. The cells were treated and incubated for 1 h at a final concentration of 0.02 µg/mL of AMB. The cells were then centrifuged and the pellet was washed thrice with 1X PBS and finally redispersed in 0.6 mL of 1X PBS, and the mean fluorescence intensity (MFI) was calculated per 10,000 cells using a BD AccuriTM C6 flow cytometer with an FLI-A channel. Each sample was run in triplicate and the data were evaluated using BD AccuriTM CFlow Plus analysis software.

### 2.12. Cellular Uptake Mechanisms in Fungal Isolates

The potential cellular uptake pathways of fluorescent design D particles were determined for clinical isolates of *C. albicans* and *C. glabrata*. The fluorescently labeled particles and cells for in vitro study were prepared as reported in Section 2.9. The cells were pre-treated with endocytosis inhibitors such as amiloride, chlorpromazine, genistein, MβCD, and nocodazole as per reported methods [26,27,28,29]. These inhibitors were added to inhibit macropinocytosis (amiloride), clathrin-mediated endocytosis (chlorpromazine), caveolae-mediated endocytosis (genistein), lipid-raft-mediated endocytosis (MβCD), and microtubule-dependent endocytosis (nocodazole). The study was conducted at 35 °C. The cells were incubated with inhibitors for one hour, followed with treatment of fluorescently labeled AMB-IONP for one hour. The control samples were prepared without using inhibitors. Cells were then centrifuged and the pellet was washed thrice with 1X PBS. At the end of three washes, the pellet was redispersed in 600 µL of 1X PBS. Each sample was subjected to flow cytometry analysis to determine MFI per 10,000 cells with the BD AccuriTM C6 flow cytometer with FL1-A channel. Each sample was run in triplicate and the data were processed with BD AccuriTM CFlow Plus analysis software. In addition, the uptake pathway was also observed with TEM and the sample preparation was the same as for the intracellular trafficking studies.

### 2.13. Intracellular Trafficking of AMB-IONP by TEM

One of the most promising methods of interpreting the intracellular localization of nanoparticles in yeast cells is by TEM [30]. The design D of AMB-IONP formulation was used against *C. albicans* and *C. glabrata* clinical isolates. Both isolates were treated with nanoparticles and incubated for 4 h at 35 °C. Following the incubation period, the isolates were washed three times with ice-cold 1X PBS and centrifuged at 13,000 rpm for 15 min. The pellet formed at the end of third wash was redispersed in 500 µL of 2.5% glutaraldehyde (electron microscope grade) in 1X PBS and incubated overnight at 4 °C. Upon the fixation of cells, the dispersion was washed thrice with 1X PBS at 200 g for 10 min each. At the end of the third wash, the cells were stained with 4% osmium tetroxide in 1X PBS for 1 h and washed an additional three times with 1X PBS and two times with distilled water. The cells were then dehydrated with 50%, 70%, 85%, and 95% ethanol for 30 min each wash.

The final dehydration was done three times with 100% ethanol. The cells were then subjected to infiltration in 1:1 ratio of Spurr’s resin and 100% ethanol, and rotated overnight. The infiltration was continued for an additional 2 h in 100% Spurr’s resin three times and the pellet was formed by centrifugation at 800× *g* for 15 min. The cells were then embedded in fresh Spurr’s resin in a casting mold and cured at 60–70 °C for 48 h. The cured block was placed in an ultramicrotome and sections of about 70 nm thick were cut with a diamond knife. Chloroform was used to smooth the sections. The sections were scooped from the knife water bath with copper 200 mesh grid and placed on filter paper and kept there until dry. The sections were stained with uranyl acetate and lead citrate to increase the contrast and electron density. The grids were observed and imaged on a JEOL 2000EX TEM (Peabody, MA, USA).

### 2.14. Intracellular Trafficking of AMB-IONP by Confocal Microscopy

To confirm the TEM results showing that the IONP were observed in endolysosomes, the cells were marked with an endolysosome-specific dye (LysoTracker^®^ deep red) [31,32]. Briefly, 600 µL of *C. albicans* and *C. glabrata* clinical isolates at a cell density of 104/mL were placed in each well for each time point. The cells were grown for an additional day to establish enough cell density for the study. This study was performed at the 4-h time point. Both isolates were treated with the design D formulation. At 15 min before the end of the time point, each well was treated with LysoTracker^®^ deep red (50 nM), a unique marker for secondary endosomes and lysosomes. Following the incubation period, the isolates were washed three times with 1X PBS and centrifuged at 13,000 rpm for 15 min. The pellet that formed at the end of the third wash was treated with one drop of Vectashield^®^ cell mounting medium with DAPI to visualize the nuclei as blue fluorescent in the images. The mixture was mixed well with a pipette and then placed on a microscopic slide and covered with a coverslip. The slide was dried in a fume hood for 15–20 min. After drying, the sides of the coverslip were immobilized to the slide with clear nail polish. The slides were again allowed to dry for another 15–20 min and stored in a freezer (−10 °C) until further use. The slides were observed with a Zeiss LSM 710 confocal laser scanning microscope (Carl Zeiss SMT Inc., USA) by using a Plan-Apochromat 60x/1.4o na Oil DIC objective lens. The obtained images were processed with Zeiss LSM Zen software 2010.

### 2.15. Determination of Tg’

Tg’, the glass transition temperature of maximally freeze-concentrated amorphous phase of the mixture of AMB-IONP and sucrose was determined by differential scanning calorimetry (DSC) with a Q2000 differential scanning calorimeter connected with the cooling system (TA Instruments, New Castle, DE, USA). The sample mixture of 10–20 µL was placed in T-Zero aluminum pans and sealed hermetically. An empty pan was prepared similarly and used as reference. The samples were equilibrated at −60 °C. After equilibration, the samples were heated at the rate of 5 °C/min to 20 °C.

### 2.16. Lyophilization of AMB-IONP

AMB-IONP (design D) were prepared as detailed in Section 2.2 and a lyoprotectant sucrose was added to the formulation. Different weight ratios of AMB-IONP to sugar were used (1:0, 1:1, 1:4, 1:8, 1:16, and 1:20). The mixture samples volumes of 500 µL were filled into 2 mL glass lyophilization vials, and rubber lyophilization stoppers were placed over the vials in such a fashion that the stoppers were partially opened to vent the moisture. The lyophilization vials with stoppers were placed into the lyophilization tray and loaded into the laboratory scale benchtop VirTis adVantage Plus lyophilizer (SP Scientific, Gardiner, NY, USA). The lyophilization cycle recipe was developed based on the Tg′ of maximally freeze-concentrated amorphous phase, previously determined by DSC. The lyophilization cycle recipe consisted of a freezing shelf temperature of −35 °C, primary drying shelf temperature of −30 °C, and secondary drying shelf temperature of 15 °C with primary and secondary drying chamber pressure (vacuum) of 100 mTorr. The lyophilizer was operated and the product temperature, shelf temperature, and condenser temperature were controlled and monitored using Wizard 2.0 software. Upon finishing lyophilization, the product mixtures in the vials were flushed with nitrogen gas at atmospheric pressure, stoppered, and sealed. The physicochemical characterization of lyophilized AMB-IONP can be found in the Supp. Section S5.

### 2.17. Short-Term Stability Studies

The aqueous dispersion and lyophilized product of AMB-IONP (design D) was subjected to a short-term stability study for up to two months. Samples were randomly selected from the batch, protected from light and placed in temperature-controlled environments under refrigerated (2–8 °C) and room temperature conditions (25 °C). Each vial was collected from each temperature-controlled environment at predetermined time points of one, two, three, four, and ten weeks and subjected to physicochemical stability analysis. Furthermore, a short-term (6–8 h) stability test for the reconstituted vials at both temperature conditions was performed. At each time point, products in the vials were visually inspected for the presence of precipitate or any abnormal occurrences. Potency testing at each sampling point and after 6–8 h on reconstituted vials stored at refrigerated (2–8 °C) and at room temperature (25 °C) conditions was conducted using a validated HPLC method. For potency testing at each time point, the free drug was removed by washing with a wash/storage buffer and subjected to magnetic separation. In addition, the samples were also tested for particle size, poly dispersity index (PDI), ζ-potential, and moisture content at predetermined time points at day 0 and at the end of ten weeks. The physical characteristics of the dispersions, including reconstituted product, were evaluated qualitatively at each sampling point. Each sample was visually inspected without magnification for changes in color, clarity, particulate matter, and product/container closure abnormalities.

## 3. Results and Discussion

### 3.1. Determination of Conjugation Efficiency

**Figure 2:**
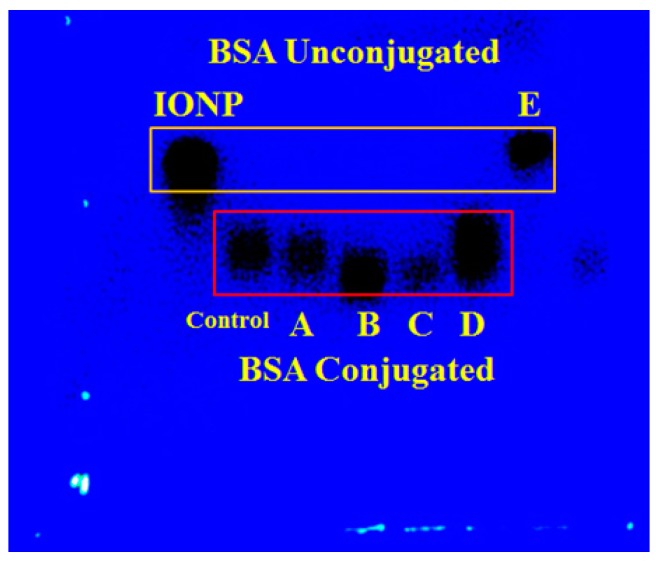
Gel electrophoresis of IONP and formulation designs Control, A, B, C, D, and E.

The conjugation efficiency of BSA to the oleic acid-coated IONP was qualitatively determined by running agarose gel electrophoresis. The unconjugated IONP were used as a control, which in general will migrate faster than the BSA-conjugated IONP [15]. It is also important to recall that the negatively charged nanoparticles should migrate towards the positive electrode and positively charged nanoparticles should migrate towards the negative electrode. The results are shown in Figure 2. The control formulation without drug and formulation designs A, B, C, and D migrated more slowly than the unconjugated formulation design E and IONP. These results confirm the successful conjugation of BSA onto the oleic acid-coated IONP.

### 3.2. Determination of Drug Loading

**Figure 3:**
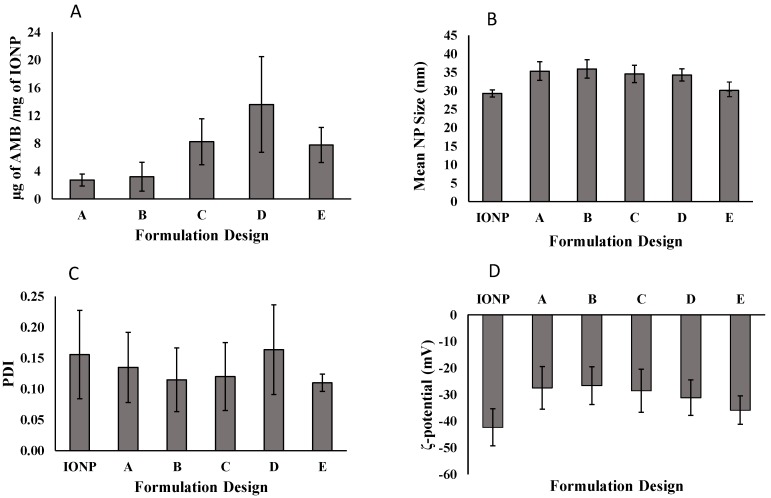
Drug loading of AMB (**A**), mean particle size of IONP (**B**), PDI of IONP (**C**), ζ-potential of IONP (**D**) for different formulation designs.

There was no set target concentration of drug for AMB-IONP formulations. We only required enough drug loaded into the IONP system to perform the job of eradicating the fungal infection. The effect of the layer-by-layer approach on the formulation designs in terms of drug loading is shown in Figure 3A. As expected, the differences in drug loading were observed with different formulation designs. In the first formulation, design A, the drug loading was found to be at the minimum for this study, with 2.7 ± 0.9 µg of AMB/mg of the nanoparticle. Design A consists of albumin conjugated to the nanoparticle cores, and then the drug is adsorbed onto this albumin layer, whereas design B consists of the attachment of AMB to the cores and then albumin is adsorbed onto the drug layer. With this approach, the drug loading was slightly increased to 3.2 ± 2.0 µg of AMB/mg of the nanoparticle. To increase the drug loading, another layer of AMB was adsorbed onto the design B, resulting in design C. As expected, the drug loading increased to 8.2 ± 3.3 µg of AMB/mg of the nanoparticle; the double layer of AMB increased the drug loading. To further increase the drug loading and to keep the formulation steps to a minimum, design D was formulated, which consists of a single coating of AMB/BSA mixture. The drug loading was further increased to 13.6 ± 6.9 µg of AMB/mg of the nanoparticle, as expected. Design E was developed as a control to other designs without attaching BSA, and the drug loading was found to be 7.8 ± 2.5 µg of AMB/mg of the nanoparticle. Considering the variability of drug loading, no statistical difference was observed between the designs C, D, and E, however, design D was selected as a lead for further studies due to (i) observed higher mean value of drug loading compared to design C and E, and (ii) lesser processing steps compared to design C.

### 3.3. Characteristics of AMB-IONP

The different formulation designs were prepared using a layer-by-layer approach. The mean hydrodynamic size of the cores, i.e., oleic acid-coated IONP, were found to be 29.3 ± 1.0 nm (Figure 3B) and in good agreement with manufacturer specifications. After the addition of additional layers onto these cores, the mean size increased up to 35 nm. The mean hydrodynamic size of the formulation designs A, B, C, D, and E were found to be 35.3 ± 2.5, 35.9 ± 2.5, 34.5 ± 2.3, 34.3 ± 1.7, and 30.1 ± 0.1, respectively (Figure 3B). There was no significant difference in mean size among the A–D formulation designs, however, design E was 14% smaller than the average hydrodynamic size of designs A–D. The obtained size range 30–35 nm is considered optimum and extremely small in nanomedicine, which is favorable for longer circulation time and greater tissue penetration. These particles can escape the reticular endothelial system (RES), and also benefit from passive targeting due to the EPR effect. The PDI is the measure of the polydispersity and it was found to be ≤0.2 (Figure 3C).

The ζ-potential of both IONP cores was found to be −42.3 ± 6.9 mV (Figure 3D) and in good agreement with manufacturer specifications. After addition of additional layers BSA and/or AMB onto these cores, the ζ-potential increased up to −27 mV. The ζ-potential of the formulation designs A, B, C, D, and E were found to be ~ −27.5 ± 8.0, −26.6 ± 7.1, −28.6 ± 8.1, −31.1 ± 6.7, and −35.8 ± 5.3, respectively (Figure 3D) indicating that these nanoparticles were negatively charged (at least above 20 mV) and will have good colloidal stability. The ζ-potential measurements before and after the coating of BSA and/or AMB showed an increase from ~ −42 mV to ~ −27 mV for all the formulation designs. This phenomenon could be attributed to the fact that both albumin and AMB are positively charged and thus add some amount of positive charge to have a net change of ζ-potential ~15 mV (from −42 mV to −27 mV).

**Figure 4:**
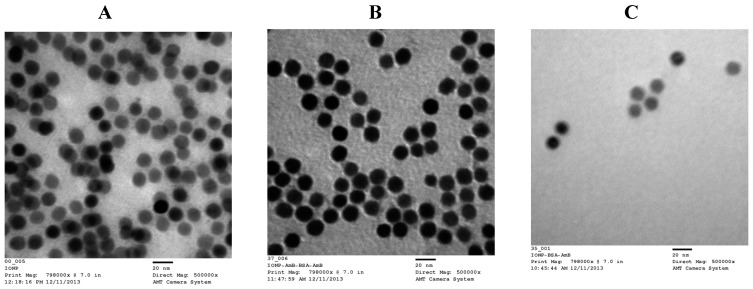
Transmission electron microscopy (TEM) image of IONP cores (**A**); design C formulation of AMB-IONP (**B**), and design D formulation of AMB-IONP (**C**) at 5 × 10^5^× magnification.

The morphological characterization of both the core nanoparticles and formulation designs of interest (C and D) were imaged by TEM with a negative staining technique. The TEM image of IONP cores is presented in Figure 4A. The image exhibited monodispersity of the nanoparticles that are spherical in shape. The size in the diameter of these cores measured by TEM is ~15 nm, which is in good agreement with the manufacturer specifications. The hydrodynamic size of oleic acid-coated IONP is between 24 and 30 nm, but the IONP cores obtained in this study are about 15 nm. In the TEM images, only the cores are denser and so clearly visible over the additional layers covering them. Not all the nanoparticles are in focus due to the multiple layers of the sample.

The TEM images of formulation designs C and D are exhibited in Figure 4B,C, respectively. These images confirm that the designs C and D formulations are monodisperse, with a spherical shape. Even though the nanoparticle size that was observed in TEM was in good agreement with that determined by the dynamic light scattering (DLS) technique, there was a slight reduction in size when measured by TEM compared to DLS. This could be attributed to the fact that the DLS provides the statistical Z-average hydrodynamic size based on the diffusion coefficient of particles in the liquid phase using the Stokes–Einstein equation, whereas TEM provides an estimation of the projected area diameter of particles in the dried state under high vacuum [33].

Thus, both studies confirm that the nanoparticles developed in this study were in the colloidal size range of 30–40 nm. This range, size, shape, and surface charge are determining factors as to whether a relatively stable liquid dispersion can be formulated for administration to the patient or presented for further dosage form development. Infected tissues become much more porous during acute inflammation and allow very small particles to enter the infection site, sometimes resulting in areas displaying an EPR effect. The entrapped particles have a great potential to become small depots for an extended or sustained release of AMB at the infection sites.

### 3.4. Drug Release Profile

**Figure 5:**
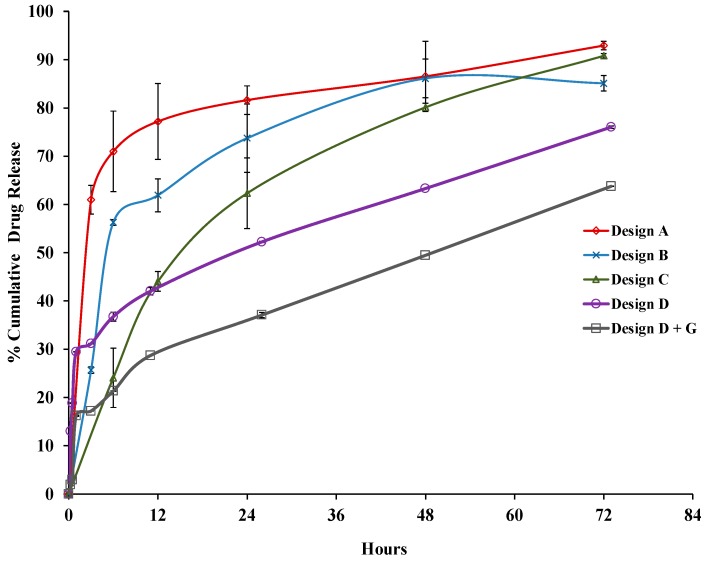
AMB release from AMB-IONP formulations.

The drug release profiles of different nanoparticles are given in Figure 5. Designs A, B, C, D, and glutaraldehyde-modified design D (D+G) showed burst releases of ~60%, ~26%, ~12%, ~31%, and ~17% at 3 h, respectively. A sustained release profile followed this determination for at least 3 days, and the cumulative drug releases at the 72 h time point were ~93%, 85%, 91%, 76%, and 64%, respectively. The high burst release for design A could be the effect of the diffusion of the drug from the surface of the albumin-coated IONP; the initial burst for other designs was much less than that of design A, as the drug was shielded by an albumin layer or a colayer of BSA+AMB in design D. The order of release of AMB was found to be A>>C>D>D+G. Designs D and D+G showed a sustained release profile, however had a higher burst than design C. Some fraction of burst release can be helpful as it leads to a high level of drug delivery at first to provide a therapeutic concentration for effective treatment following a sustained release.

The cross-linking of albumin with glutaraldehyde has given rigid structure that delays the diffusion of the drug from the nanoparticles. Hence, kinetics of a more sustained release character were observed in D+G compared to design D. Data for the AMB release from formulation designs A, B, and C of AMB-IONP were analyzed for release kinetics by zero-order (cumulative %drug release versus time), first-order (log cumulative %drug release vs. time), Higuchi (cumulative %drug release vs. square root of time), Hixson–Crowell (cube root of %drug release vs. time), and Korsmeyer–Peppas (log cumulative %drug release vs. log time) models [34,35,36]. Based upon the value of the release exponent (*n*), which is the slope of log % cumulative release vs. log time, and the highest regression coefficient (*r^2^*) value among the models (Supp. Table S1), the major mechanism of drug release from the formulations was found to be Fickian diffusion with first order and Higuchi kinetic models as best fit.

### 3.5. Colloidal Stability in FBS

**Figure 6:**
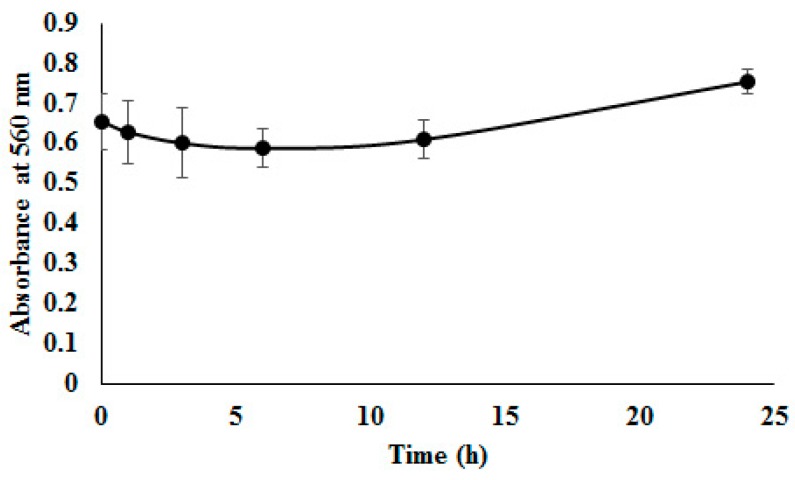
Physical stability of design D formulation of AMB-IONP in 50% fetal bovine serum (FBS). (The data were presented as mean ± SD (*n* = 3). Particles retained their physicochemical properties without aggregation for a prolonged period of time (24 h)).

Stability determination of one AMB-IONP formulation (design D, selected as the lead formulation due to its higher drug loading) in serum was considered essential for in vivo usage, where dilution after intravenous administration could result in nanoparticulate aggregation, disassembly, and/or drug leakage. AMB-IONP were incubated in FBS (50%) at 37 °C with gentle shaking. The samples were then analyzed for 560 nm absorbance. The absorption method was used as AMB-IONP cannot be accurately detected in dense serum solution by the DLS technique, due to the matrix protein interference from serum [37]. Aggregation phenomena were characterized by increased absorbance to higher values. The interaction of AMB-IONP with various serum proteins in the incubation media could lead to neutralization of the ζ-potential of nanoparticles. The process could potentially cause the reduction of repulsion energy among nanoparticles, and gradually facilitate an agglomeration process, which could lead to the formation of larger particles and instability to disperse. AMB-IONP was found to be stable in serum, retaining its integrity up to 24 h as observed in Figure 6. The data also indicate no significant changes in absorbance values at 560 nm.

### 3.6. Cellular Uptake of AMB-IONP

Both the qualitative and quantitative analysis of the uptake of the design D formulation of AMB-IONP was determined with confocal laser scanning microscopy and flow cytometry, respectively. Confocal microscopy was employed to visualize the localization of nanoparticles in the cellular compartments, while flow cytometry was employed to determine the MFI for clinical isolates of *C. albicans* and *C. glabrata* treated with the formulation. The AMB-IONP were labeled with coumarin 6 dye by 1% weight of IONP for visualization and MFI determination. The fluorescent-labeled AMB-IONP were also subjected to physicochemical characterization (size, PDI, and ζ-potential), and the results obtained were similar to those of non-fluorescent labeled AMB-IONP (data not presented).

**Figure 7:**
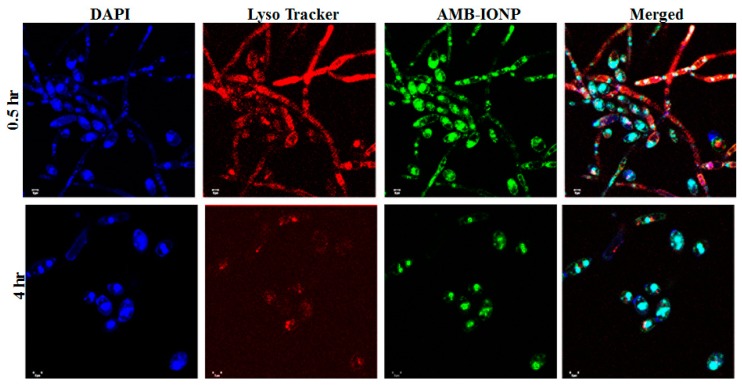
Confocal scanning laser microscopy images of cellular uptake of design D in *C. albicans.* (magnification 60×). The nucleus was stained blue with DAPI. The images indicate the endolysosomal localization with colocalization of AMB-IONP (green) and lysotracker in red in the merged image and time-dependent uptake of nanoparticles in green, with a maximum uptake at 0.5 h. The scale bar represents 2 µm.

**Figure 8:**
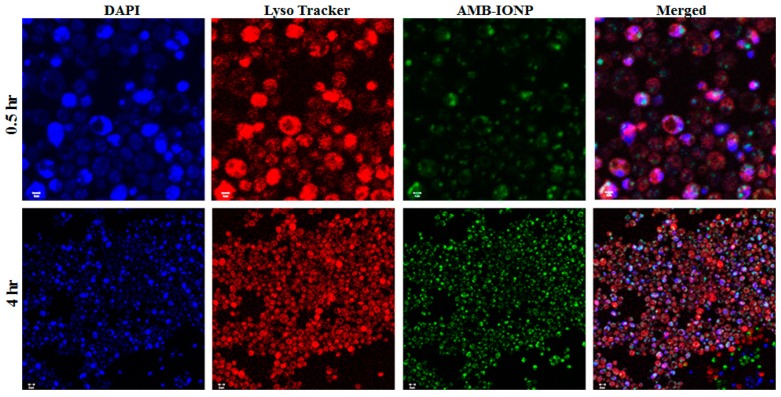
Confocal scanning laser microscopy images of cellular uptake of design D in *C. glabrate*. (magnification 60×) The nucleus was stained blue with DAPI. The images indicate the endolysosomal localization with colocalization of AMB-IONP (green) and lysotracker in red in the merged image and time-dependent uptake of nanoparticles in green, with maximum uptake at 4 h. The scale bar represents 1 µm for 0.5 h and 2 µm for 4 h.

The images of confocal microscopy for the cellular uptake of the design D formulation in the clinical isolates of *C. albicans* and *C. glabrata* are represented in Figure 7 and Figure 8, respectively. Coumarin 6 (indicative of AMB-IONP), LysoTracker^®^ (indicative of secondary endosome and lysosome), and DAPI (indicative of nuclei) fluoresce green, red, and blue, respectively. The confocal microscopy data revealed that the AMB-IONP were co-localized with endosomes and lysosomes and were widely existing in the cytoplasm of both isolates. For *C. albicans*, the 0.5-h sample exhibited significantly higher uptake when compared to the 4-h time point. Whereas, for *C. glabrata*, the 4-h time point exhibited significantly higher uptake in comparison to the 0.5-h time point.

**Figure 9:**
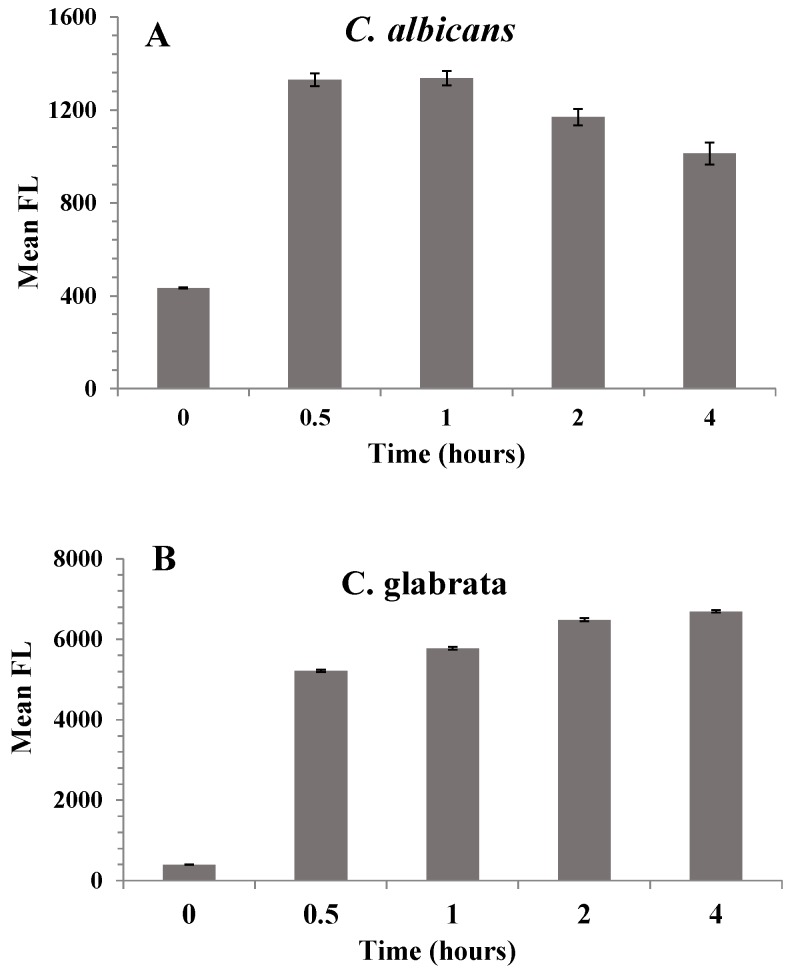
Cellular uptake of design D in clinical isolates *C. albicans* (**A**) and *C. glabrata* (**B**) by flow cytometry.

Flow cytometry revealed the time-dependent cellular uptake of nanoparticles in both the isolates tested. The MFI indicated maximum uptake within the first 30 min after treatment for *C. albicans* as shown in Figure 9. The uptake plateaued between 0.5 and 1 h after treatment. The *C. glabrata* isolate showed that the maximum uptake was at the 4-h time point. The uptake plateaued between 2 and 4 h after treatment. These results were in good agreement with confocal microscopy data. Thus, the confocal microscopy and flow cytometry results confirm the uptake of AMB-IONP in both the isolates tested.

### 3.7. In Vitro Efficacy of AMB-IONP Fungal Clinical Isolates of Candida Species

**Figure 10:**
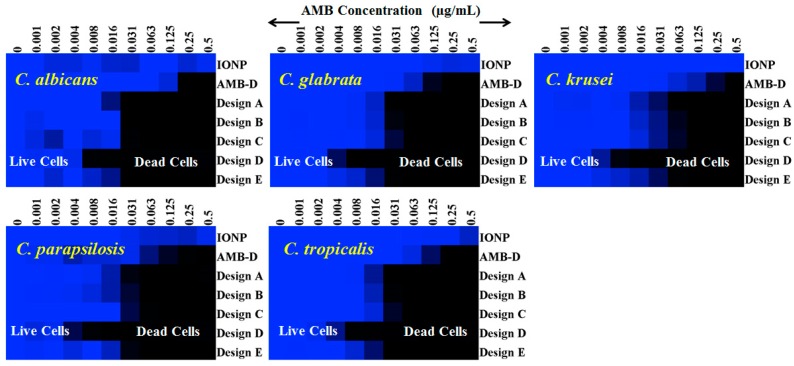
Representative heat maps for susceptibility testing of indicated *Candida* isolates. For control without AMB (IONP) the concentrations indicate the same amount of particles treated for other formulation designs. Each well was normalized to the no drug growth control. The data are representative of at least two reproducible experiments.

**Figure 11:**
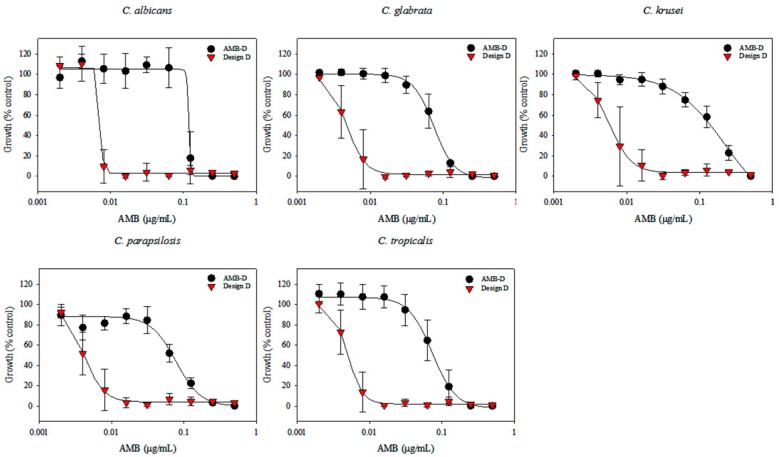
Dose response curves for treatment of clinical isolates of the indicated species of *Candida* (*n* = 3). Cell densities were measured at 48 h and normalized to the control wells containing no drug.

**Figure 12:**
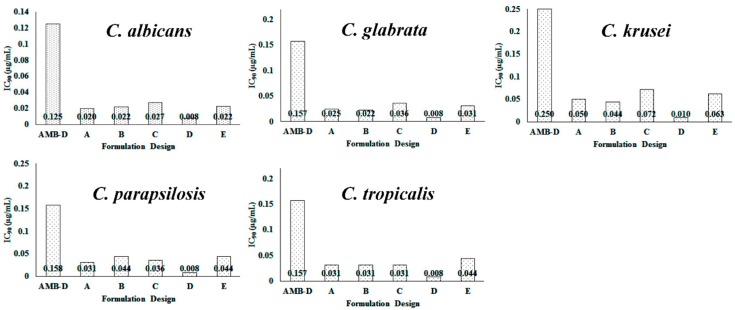
IC_90_ values estimation for susceptibility testing in fungal clinical isolates. The data presented as geometric mean for AMB-D, design A, B, C, D, and E with *n* = 3, 3, 2, 5, 3, and 2, respectively. The IC_90_ value for control formulation (IONP without AMB) was found to be >3 µg/mL.

Susceptibility testing was performed once the cellular uptake of these AMB-IONP formulations was confirmed. Representative heat maps and dose-response curves for the susceptibility testing are shown in Figure 10 and Figure 11, respectively. The control formulation that contains no AMB showed no efficacy or toxicity towards the fungal clinical isolates tested. This indicates that the amount of IONP used can be considered safe for the study. The percentage of dead cells were higher for the formulation design D. The IC90 values calculated at the end of 24 h of the study, presented in Figure 12, indicated that all the formulation designs of AMB-IONP had better killing power over the marketed conventional product AMB-D against *C. albicans C. glabrata, C. krusei, C. parapsilosis*, and *C. tropicalis*. Among the formulations developed for this study, design D was found to be the lead formulation with 16–25-fold more efficacy in eradicating 90% of any discernible growth when compared with AMB-D.

There could be many reasons for better efficacy of the developed formulation designs, particularly for lead design D, which was also found to be the lead in the amount of drug loading. First, these nanoparticles are extremely small (30–40 nm) for nanomedicine, and demonstrate higher cellular uptake, either due to a targeting effect or due to their smaller sizes when compared to the AMB-D micellar product. Second, the albumin-bound AMB to the BSA can act as a targeting agent as well as an anti-infective due its higher binding affinity towards the ergosterol present in fungal cell membranes. Third, small particles with a ≤40 nm size with the concomitant larger surface area, and having albumin coating as a targeting agent, can potentially have an additive effect, producing a higher cellular uptake.

As each particle is loaded with a significantly high payload of AMB, which is delivered at/inside the fungal cell membrane, this may explain the relatively higher killing effectiveness. More AMB means a better chance for wider pores, leading to higher efficiency in killing fungal cells [38]. The additive effect of size in terms of cellular uptake can also be seen in the fungal isolates of *C. albicans* CA1008 and Calowa60, which contain less than 1% of ergosterol. Formulation design D was found to be more efficacious than AMB-D, but overall required twice the AMB amount to kill fungal cells with low ergosterol (data not presented). This also confirms the targeting ability of the AMB on formulation designs for other fungal clinical isolates tested. Overall, these results confirm that the lead formulation was design D with up to 25-fold more efficacy than AMB-D.

**Figure 13:**
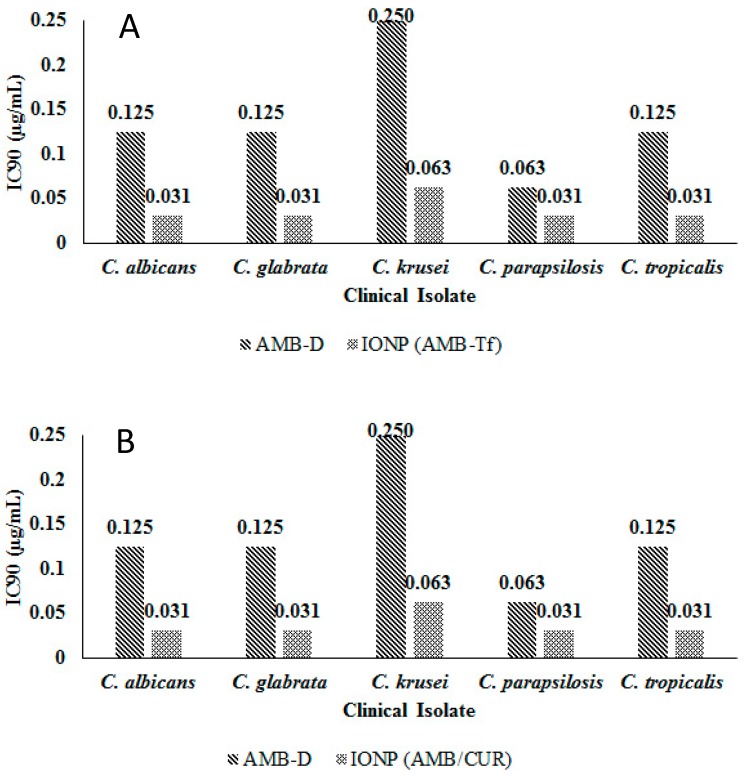
IC_90_ values estimation for susceptibility testing of indicated *Candida* clinical isolates treated with AMB-D and transferrin (Tf)-bound design D (*n* = 1) (**A**), and AMB-D and dual drug loading of AMB and curcumin (CUR) in design D (*n* = 1) (**B**).

The transferrin-bound design D formulation has significantly lower IC90 values over AMB-D for clinical isolates tested (Figure 13A). This small feasibility study results (*n* = 1) indicated that transferrin can also potentially be used as targeting ligand, particularly for targeting CNS, where transferrin receptors are overexpressed during inflammation or tumor growth [39]. Similar results were observed with a dual drug loading formulation, with AMB and curcumin (Figure 13B). The outcome of this study suggests that this formulation design could be used to load multiple drugs that have different binding domains on the albumin layer.

### 3.8. Cell Association Study

**Figure 14:**
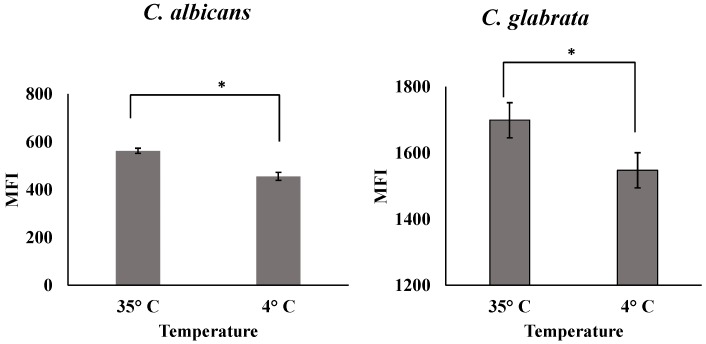
Cell association study of AMB-IONP (mean ± SD; *n* = 3; * *p*-value of < 0.01, *t*-test).

The results of cellular association studies at 35 °C and 4 °C for the *C. albicans* and *C. glabrata* clinical isolates are presented in Figure 14. The MFI reflects the uptake of the nanoparticles into the cells. The results indicated that uptake was reduced in both isolates when the study was conducted at 4 °C compared to 35 °C. Receptor-mediated endocytosis is an energy/temperature dependent pathway, which requires ATP for significant function. The reason for the reduced uptake at 4 °C could be that the uptake mechanism is receptor-mediated endocytosis that requires energy. The uptake mechanism at 4 °C was reduced and may be stopped due to the receptor-mediated nanoparticles being internalized before washing with PBS. These results suggest that the uptake pathway mechanism of AMB-IONP into both *C. albicans* and *C. glabrata* clinical isolates was controlled by receptor-mediated endocytosis.

### 3.9. Uptake Mechanism of AMB-IONP into Cells

**Figure 15:**
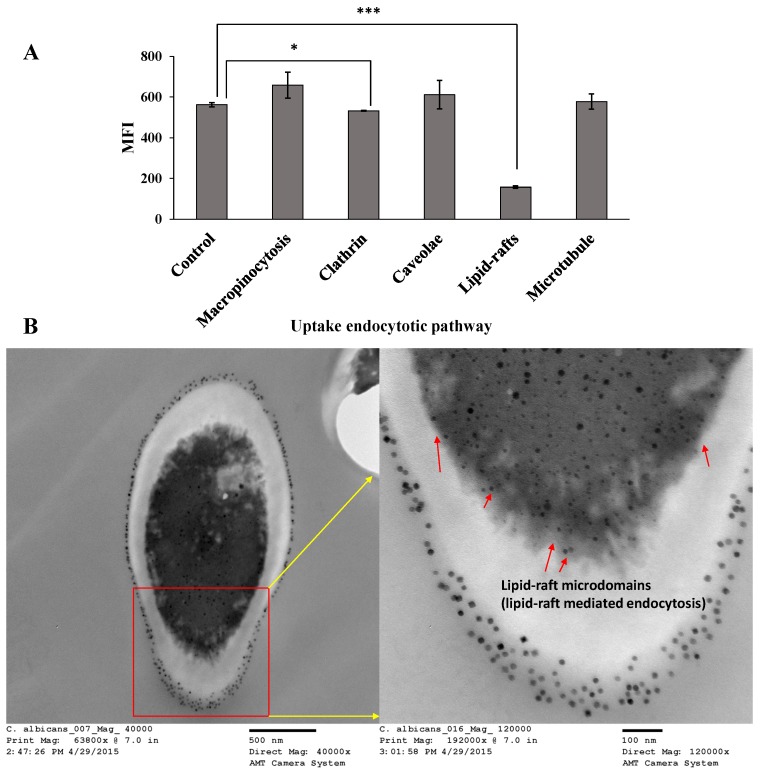
Cellular uptake mechanism study (**A**) and TEM Images (**B**) in *C. albicans* treated with AMB-IONP (Mean ± SD, *n* = 3; * *p* < 0.05, *** *p* < 0.001, *t*-test).

**Figure 16:**
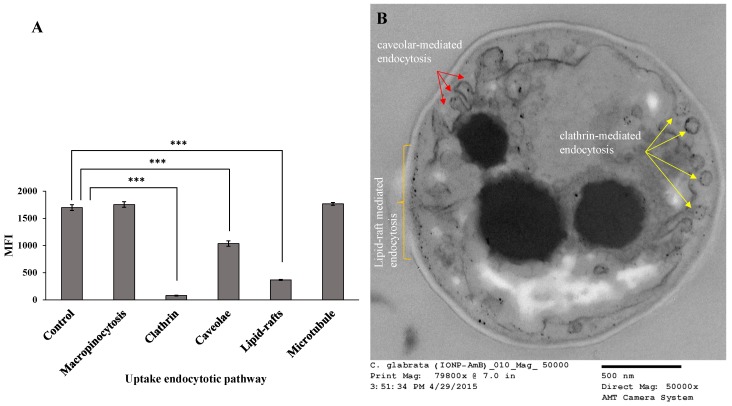
Cellular uptake mechanism study (**A**) and TEM Images (**B**) in C. glabrate treated with AMB-IONP (Mean ± SD, *n* = 3; * *p* < 0.05, *** *p* < 0.001, *t*-test).

The cellular uptake pathway of nanoparticles mediated through endocytosis can include micropinocytosis, clathrin-mediated endocytosis, caveolae-mediated endocytosis, clathrin/caveolae independent endocytosis, lipid-raft-mediated endocytosis, and uptake through microtubules [26,27,28,29,40]. A schematic representation of these different mechanisms is given in Supp. Figure S2. The flow cytometry results of the effect of endocytosis inhibitors on the uptake of fluorescently labeled AMB-IONP in both clinical isolates of *C. albicans* and *C. glabrata* are presented in Figure 15A and Figure 16A, respectively.

Even though the data indicated a significant difference in MFI of clathrin-mediated endocytosis inhibitor and lipid-raft-mediated endocytosis when compared to the control group, the major pathway for uptake of AMB-IONP into *C. albicans* was determined to be lipid-raft-mediated endocytosis (Figure 15A). Lipid raft endocytosis is defined as an endocytic pathway, which is sensitive to cholesterol discomposure [41]. In yeast eukaryotes, cell receptor and fluid phase endocytosis is reliant on the sterol found in the yeast cells, ergosterol. Ergosterol is the major sterol found in the yeast plasma membrane. This strongly suggests a pathway similar to the cholesterol-dependent endocytosis in mammalian cells. This is reinforced by the fact that ergosterol has a greater ability to form lipid rafts than does cholesterol [42]. MβCD is an agent used to extract and/or sequester membrane sterols [29,43]. As MβCD inhibited the lipid-raft-mediated endocytosis pathway, very few particles gained entrance into the cell. Therefore, it is concluded that lipid-raft-mediated endocytosis is the major pathway for uptake of AMB-IONP into *C. albicans.*

It was already known that AMB has a greater binding affinity towards the ergosterol found in the fungal cell membrane. Hence, these data also suggest that the AMB is on the surface of the design D formulation of AMB-IONP, which helped target the nanoparticles to the fungal isolate tested, and achieve a more efficacious delivery system over AMB-D. Additionally, the uptake mechanism is also observed with TEM and the image of *C. albicans* cells incubated with AMB-IONP for 4 h revealed lipid-raft microdomains as seen in Figure 15B.

The nanoparticles were found to have adhered to the fungal cell wall to get into the cell membrane, to bind and/or extract ergosterol from the lipid bilayer of the membrane as per the proposed mechanism of action of AMB. The nanoparticles were present in the cell wall, but TEM could not identify them due to its dense structural composition. The particles were also found in abundance at the cell membrane as seen in Figure 15B indicate the possibility of the lipid-raft-mediated endocytosis pathway for cellular uptake. The morphology of the clathrin- and perhaps caveolar-independent carriers was found to be similar to the observations reported by M. Kirkham and R.G. Parton [41]. This pathway lacks study and needs to be further characterized in detail.

For the *C. glabrata* clinical isolate, there were multiple major uptake pathways of nanoparticles, which included clathrin-mediated endocytosis, caveolar-type endocytosis, and lipid-raft-mediated endocytosis (Figure 16A). Chlorpromazine is used to block the formation of clathrin-coated pits at the plasma membrane and leads to assembly of clathrin lattices on endosomal membranes [44]. Treatment with chlorpromazine exhibited a significant difference in uptake of the particles, leading to the determination that this was a major pathway for endocytosis. Caveolae-mediated endocytosis comprises the development of 50–100 nm invaginated plasma membranes, which are identified as caveolae [45]. The main composition of caveolae includes caveolin, glycolipids, and cholesterol. Genistein is a specific inhibitor of caveolae-mediated uptake [46]. Genistein showed a significant inhibition of cellular uptake of AMB-IONP in *C. glabrata*, leading to another possible uptake mechanism. The caveolae is formed when caveolin-1 and caveolin-3 are expressed in non-muscle cells and muscle cells, respectively [41]. It was recognized as a specific form of lipid raft domain, which has distinctive morphology created by caveolin. It was found that caveolin-1 directly interacts with membrane sterols, and upon membrane, sterol depletion leads to disruption of caveolae. Thus, both caveolar-mediated endocytosis and lipid-raft-mediated endocytosis are affected by ergosterol depletion caused by MβCD inhibitor. Together, these results suggest that the possibility of multiple endocytic mechanisms in yeast cells. These findings are in agreement with the literature concerning the possibilities of multiple endocytic mechanisms in yeast cells [41].

Additionally, the uptake mechanism was observed with TEM and the image of *C. glabrata* cells incubated with AMB-IONP for 4 h revealed clathrin-mediated endocytosis, caveolar-type endocytosis, and lipid-raft-mediated endocytosis as represented in Figure 16B. The first mechanism, clathrin-mediated endocytosis, was clearly observed with TEM as the image of clathrin-coated structures found with vesicular morphology and the size (~65 nm) of the vesicles are in agreement with the literature [43,47]. The IONP were found along the peripheral and inside of the vesicles. This interpretation confirmed that clathrin-mediated endocytosis was one of the cellular uptake pathways for these nanoparticles.

The TEM image also provided evidence of lipid-raft-mediated endocytosis as a second cellular uptake pathway. The particles found in abundance at the cell membrane indicated the possibility of the lipid-raft-mediated endocytosis pathway for the cellular uptake. The TEM image also revealed the evidence of caveolae-mediated endocytosis as a third uptake mechanism. The caveolae-coated pits are found with IONP particles and the morphology was vesicular/tubulovesicular with a size of 50–100 nm in diameter, which is in agreement with the literature [48]. The electron-dense particle size of IONP cores was also found to agree with manufacturer specifications (~15 nm). The TEM findings agree and complement the results of the uptake mechanism study with inhibitors.

Since there were three possible cellular uptake pathways available for *C. glabrata*, the nanoparticles were infrequently seen on or near the cell wall and cell membrane after 4 h of incubation when compared to *C. albicans*. For *C. albicans* there is only one major pathway available for uptake of particles, which could delay the uptake of nanoparticles into the cells. This could be a reason for the better efficacy of design D in *C. glabrata* over *C. albicans*. The MIC data indicated that the design D was nine-fold more efficacious than AMB-D in *C. glabrata*, whereas it was only seven-fold more efficacious in *C. albicans* when compared to AMB-D.

### 3.10. Intracellular Trafficking of AMB-IONP in Fungal Clinical Isolates

**Figure 17:**
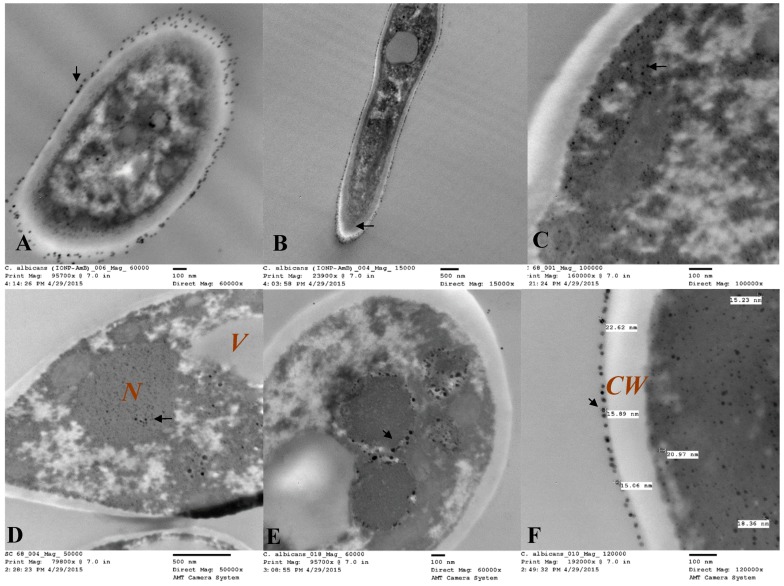
Intracellular trafficking localization of AMB-IONP in *C. albicans* by TEM. The TEM images revealed that the nanoparticles were localized at or near the cell wall (images **A** and **F**) and membrane (images **A**, **B** and **F**) and inside the cytoplasm (imges **C** and **F**), nucleus (image **D**), and endolysosomal vesicles (images **D** and **E**), as indicated by arrows in images shown at different maginication **A** (60 × 10^3^), **B** (15 × 10^3^), **C** (100 × 10^3^), **D** (50 × 10^3^), **E** (60 × 10^3^) and **F** (120 × 10^3^). The images A and D indicates a dying cell with disorganization of intracellular components.

**Figure 18:**
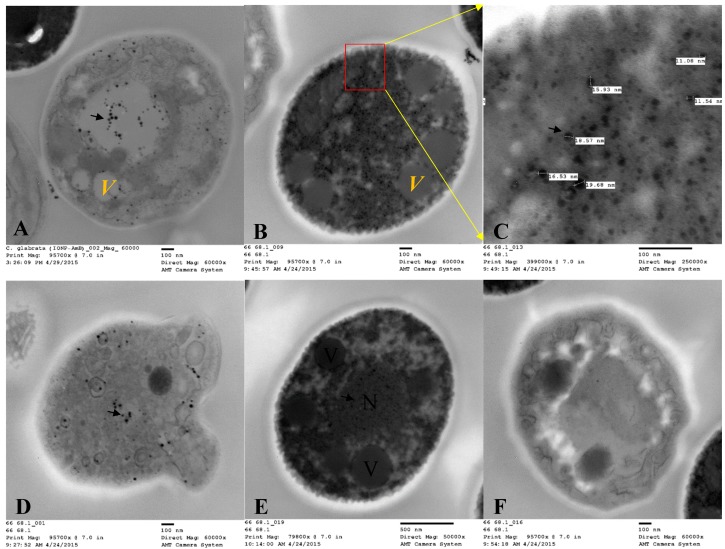
Intracellular trafficking localization of AMB-IONP in *C. glabrata* by TEM. The TEM images revealed the nanoparticles were localized at or near the cell wall and membrane (image **A**) and inside the cytoplasm (images **B** and **C**), nucleus (images **D** and **E**), and endolysosomal vesicles (image **E**), as indicated by arrows in images shown at different magnification **A** (60 × 10^3^), **B**(60 × 10^3^), **C**(250 × 10^3^), **D**(60 × 10^3^), **E** (50 × 10^3^) and **F** (60 × 10^3^). The image F indicates a dying cell with disorganization of intracellular components.

For both clinical isolates of *C. albicans* and *C. glabrata*, in vitro cellular internalization, localization, and intracellular trafficking were explored by TEM. Figure 17 and Figure 18 represent the TEM images for *C. albicans* and *C. glabrata* clinical isolates, respectively, which were incubated with AMB-IONP for 4 h. IONP are easily traceable with TEM imaging. TEM images revealed the nanoparticles were localized at or near the cell wall and cell membrane and inside the cytoplasm, nucleus and endolysosomal vesicles for both isolates tested.

Through TEM images, one can also derive additional information related to the actions of a delivery system on the cells upon internalization. After 4 h of treatment, the TEM images demonstrated the disorganization of the cytoplasm with deformed nuclei and other intracellular components, including the endoplasmic reticulum and mitochondria (Figure 17 and Figure 18). Also observed was a significant increase in the number of vacuoles [49]. The increase in vacuole number could be correlated to the actions of AMB, that tends to extract ergosterol. Toxic ergosterol precursors could possibly accumulate in the vacuoles. The images also revealed the disintegration of the cell wall and the cytoplasmic membrane along with cytoplasm leakage. These findings indicate dying cells (see Figure 17D and Figure 18F).

Confocal microscopy fluorescence provided evidence of the colocalization of fluorescence signals from nuclei (blue), endosomes and lysosomes (deep red), and fluorescently labeled AMB-IONP (green). After 4 h of incubation of clinical isolates of *C. albicans* and *C. glabrata* with the nanoparticles, Supp. Figure S3 shows colocalization, which agrees and complements the results of the TEM images.

### 3.11. Tg’ Determination for the Development of Lyophilization Cycle

The stability of AMB in dispersion form during storage is a major challenge in the development of IONP formulations. Physical instability leads to leakage of loaded drug and aggregation of particles resulting in increased particle size [50]. The physicochemical stability can be improved by removing water from the formulation by lyophilizing the product. Water tends to crystallize during the freezing step resulting in ice crystals, during which the nanoparticles and solute show a tendency for fusion and aggregation due to their higher concentration at this stage.

To avoid this problem, lyoprotectant sugar can be added. The sugar has a capability of forming an amorphous matrix around the particles that protect from fusion and prevent the rupture of the particles due to the crystallization of ice [51,52]. Even though trehalose is the most widely used lyoprotectant, reports indicate that sucrose has better control over the nanoparticle size than trehalose [50,53]. Additionally, trehalose can also undergo crystallization during lyophilization. The integrity of the nanoparticles could be compromised due to crystallization during lyophilization. Thus, trehalose was not used and sucrose (non-crystallizing solute) was evaluated as a lyoprotectant in an effort to develop a lyophilized AMB-IONP formulation.

The glass transition temperature (Tg’) of the maximally frozen concentrated mixture of the formulation recipe is an important factor in determining the freezing and primary drying temperatures when developing a lyophilization process. To dry the formulation product without losing its structural properties, the formulation dispersion should be maintained in the solid-state below the glass transition (collapse) temperature. The Tg’ was determined using DSC, using incremental weight ratios of IONP to sucrose as lyoprotectant. The DSC thermograms of samples with different sucrose ratios to IONP are exhibited in Supp. Figure S4. The data indicated that the Tg’ values increased directly with a greater sucrose weight ratio in the mixture [54]. The Tg’ value was found to be about −25 °C. Thus, the primary drying temperature was optimized to 2–5 °C < Tg’ to inhibit collapse.

The temperature of −35 °C was found to be optimum for a freezing temperature during the lyophilization process with the cooling rate of ~0.4 °C/min and then held at this temperature for 6 h. The primary drying temperature of −30 °C was found to be optimum to avoid the collapse of the cake. The end of the primary drying cycle was determined by the increase in product temperature that matched the shelf temperature of the lyophilizer. The total hold time of 12 h at −30 °C was applied. The chamber pressure of 100 mTorr was used, which is below the vapor pressure of ice at sub-zero temperatures [55,56]. The main reason for the sublimation process during the primary drying cycle was the difference in the ice vapor pressure at −30 °C and the chamber pressure of the lyophilizer. The temperature of 15 °C was used as the secondary drying temperature and held for 6 h at that temperature with a maximum vacuum of 100 mTorr. The product was them removed from the lyophilizer, flushed with nitrogen gas, and sealed.

### 3.12. Visual Inspection and Physicochemical Characterization of Lyophilized AMB-IONP

The products with different sucrose weight ratios were visually inspected to determine the cake height, any cake separation, product on rubber closures, and the presence of any surface abnormalities, such as air bubbles or puffing. The lyophilized product with no sucrose exhibited an unacceptable meltback cake Supp. Figure S5. The products with sucrose exhibited no cake separation, no product on the rubber stoppers, and there were no visible surface abnormalities. However, there was a significant change in the cake height to the initial fill volume when the sucrose weight ratio of 8 or lower was used. The reduction in cake height is significant, resulting in poor and unacceptable cakes. The sucrose weight ratio of 16 and higher was found to be optimum without any visible abnormalities. In addition, the lyophilized product was found to be instantaneously redispersed upon reconstitution with water.

The mean size, PDI, and zeta potential of AMB-IONP were found to be retained after lyophilization at 1:16 weight ratio of IONP to sucrose (Table 1). At lower sucrose ratios, the particle size and zeta potential were slightly changed, suggesting that fusion and aggregation of IONP occurred during lyophilization or this may be attributed to the surface adsorption by sucrose molecules. Overall, although the physicochemical properties of the nanoparticles were retained at lower sucrose ratios, a reduction of the cake height to initial fill volume was obtained. One of the critical quality attributes for any lyophilized product is the amount of the residual moisture content. In general, a higher moisture content potentially leads to poor stability with loss of therapeutic activity [57]. The lower residual moisture content of 0.5–3% w/w for lyophilized products containing either small molecular weight drugs or molecules with significantly higher molecular weights is recommended [58]. Karl Fisher titration analysis revealed a moisture content of <1.5% w/w for the lyophilized product of AMB-IONP containing sucrose (1:16), which is considered optimum for long-term storage and was within the acceptable limits set by the FDA [59]. Thus, a weight ratio of 16:1 sucrose to IONP was selected as optimum for the lyophilization formulation, based on visual inspection and physicochemical characterization.

### 3.13. Stability of Aqueous Dispersion and Lyophilized AMB-IONP

**Figure 19:**
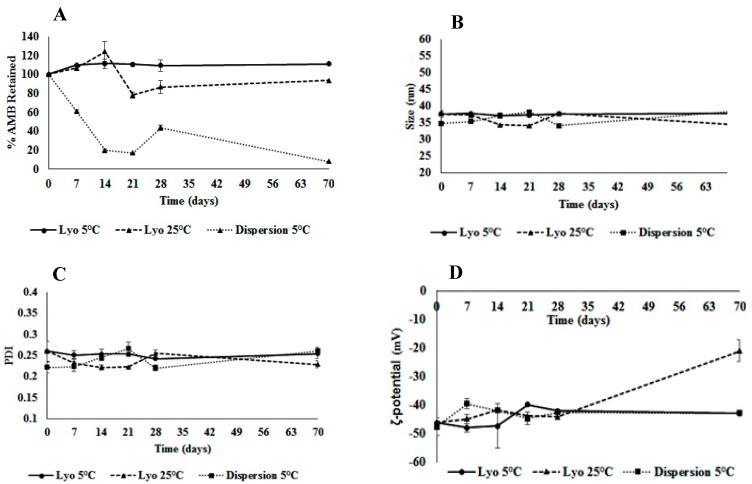
Short-term stability of active pharmaceutical ingredients (APIs) (**A**), size (**B**), PDI (**C**), and zeta-potential (**D**) in lyophilized and liquid dispersion formulations (mean ± SD, *n* = 3).

The stability of the liquid dispersions and lyophilized formulations of AMB-IONP (design D) were evaluated in 2 cc glass vials (protected from light) at 2–8 °C (refrigerated condition) and 25 °C (room temperature condition) for up to two months. At each predetermined time point, the physicochemical characters such as potency, mean particle size, PDI, and ζ-potential were determined. The stability data for 2–8 °C and 25 °C are presented in Figure 19. The liquid dispersion stored at ambient conditions of 25 °C showed significant degradation by the end of the second week of storage and was discontinued from the study. The lyophilized product stored at 2–8 °C exhibited good stability with more than 90% of drug retained even after ten weeks. The next most stable product was the lyophilized product stored at 25 °C, with up to 94% of drug retained in ten weeks. The liquid dispersion product stored at 2–8 °C did not exhibit good stability with only 8% drug retained by end of the study.

These results indicate that AMB is sensitive to temperature and exhibits thermal degradation, which is in good agreement with a previous report by Manosroi et al. in 2004 [60]. The drug is more stable in the dry form in lyophilized cakes rather than in aqueous medium. Removal of water from the liquid dispersion through lyophilization improved the shelf life by preventing drug degradation through hydrolysis or oxidation. In addition, the drug might be partitioning out from nanoparticles in the aqueous phase compared to the lyophilized form.

All the physicochemical characters of the AMB-IONP in both forms stored at both temperatures retained the mean particle size, PDI, and ζ-potential without any significant changes and were within the acceptable limits. Visual inspection could not identify any physical abnormalities at each time point. The moisture content of the lyophilized cake was maintained below 1.5% w/w throughout the testing period. The reconstituted lyophilized product was found to be stable at ambient conditions for up to 7 h (data not presented). The lyophilized AMB-IONP might be the best formulation for AMB, due to its higher stability when stored at 2–8 °C, than the liquid dispersion form stored under room temperature conditions.

## 4. Conclusions

In summary, a targeted drug delivery system (AMB-IONP) of AMB was successfully designed and developed using a layer-by-layer approach. The design D approach with the order of the layers IONP, then AMB bound BSA (from inside to outside) was found to be promising, with maximum drug loading of 13.6 ± 6.9 µg of AMB/mg of nanoparticle. The size and ζ-potential for all the formulation designs were found to be optimum for a nanomedicine with ≤36 nm and ~ −20 mV, respectively. The PDI was also found to be optimum with ≤0.2 PDI. The drug release profile indicated a burst release for up to 3 h, followed by a sustained drug release profile lasting up to at least 72 h. The design D formulation was stable in 50% serum for up to 24 h.

The flow cytometry results for cellular uptake of formulation design D in *C. albicans* and *C. glabrata* clinical isolates exhibited time-dependent uptake. The cellular uptake reached a plateau between 0.5 and 1 h and between 2 and 4 h for *C. albicans* and *C. glabrata*, respectively. All of the formulation designs of AMB-IONP exhibited improved efficacy over marketed conventional AMB-D product in susceptibility testing conducted in clinical isolates of *C. albicans* SC5314, *C. glabrata* 66032, *C. krusei* CK1, *C. parapsilosis* CP1, and *C. tropicalis* CT1. Design D was found to be superior and was 16–25-fold more efficacious than the conventional product. Even in clinical isolates of *C. albicans* CA1008 and Calowa60 that have < 1% of ergosterol, design D had better kill power over AMB-D.

The endocytosis inhibitor evaluation and TEM images of IONP revealed that the major particle uptake pathway for *C. albicans* was lipid-raft mediated endocytosis, whereas for *C. glabrata* clinical isolate, the major uptake mechanisms were multiple and included clathrin-mediated endocytosis, caveolar-type endocytosis, and lipid-raft-mediated endocytosis. TEM images provided evidence that the AMB-IONP were localized at or near the cell wall and membrane and inside the cytoplasm, nucleus, and endolysosomal vesicles for both isolates tested. The confocal study confirmed the colocalization of fluorescence signals from nuclei, endosomes, lysosomes, and fluorescently labeled AMB-IONP, 4 h after treatment. The intracellular trafficking results suggested that the AMB-IONP-targeted drug delivery system successfully delivered AMB payload at and/or inside fungal cells, leading to potential therapeutic advantages over AMB-D.

Finally, a stable lyophilized formulation of AMB-IONP was successfully prepared with the selection of an appropriate amount of the lyoprotectant sucrose. With this design, the layering process and eventual small-scale production can be greatly simplified for animal studies and clinical trials. Therefore, it is expected that these processes and properties can be more amenable to production scale-up, however, more process development studies are needed. The designed nanoparticles have the potential to be further developed into robust nanotheranostics for targeted drug delivery.

## Figures and Tables

**Table 1:** Characterization of lyophilized AMB-IONP with different weights of sucrose.

Parameter	Before Lyo (Control)	After Lyo (Sucrose Weight Ratio to the Weight of IONP)				
		20	16	8	4	1
**Size (nm)**	33.8 ± 1.02	35.0 ± 0.12	34.5 ± 0.07	36.4 ± 0.69	39.0 ± 0.21	36.9 ± 0.04
**PDI**	0.20 ± 0.01	0.16 ± 0.02	0.14 ± 0.01	0.20 ± 0.01	0.26 ± 0.02	0.22 ± 0.01
**ζ-potential (-mV)**	22.3 ± 5.4	33.9 ± 0.9	25.1 ± 1.5	13.3 ± 4.7	31.3 ± 0.9	35.1 ± 2.2

The data are presented as mean ± SD, *n* = 3.

## Supplementary Materials

The following are available online at https://www.mdpi.com/1999-4923/12/3/247/s1, Figure S1: Schematic diagram of the in-house experimental setup for in vitro drug release study, Figure S2. Schematic representation of different cellular uptake pathways, Figure S3. Intracellular trafficking localization of AMB-IONP in *C. albicans* and *C. glabrata* by confocal microscopy, Figure S4. DSC thermograms of different sucrose weight ratios to IONP, Figure S5. Physical appearance of lyophilized product of AMB-IONP with different weight ratios of sucrose. Table S1. In vitro release kinetics parameters of AMB from the IONP formulations. Procedural details about Formulation Preparation (section S1), Drug loading testing (section S2), Particle size and zeta potential testing (section S3), Physicochemical characterization of lyophilized IONP (section S5).

## Abbreviations

AMB: Amphotericin B deoxycholate; AMB: Amphotericin B; AMB-IONP: Amphotericin B loaded Iron oxide nanoparticle; BSA: Bovine serum albumin; DAPI: 4’,6-diamidino-2-phenylindole; DLS: Dynamic light scattering; DMSO: Dimethyl sulfoxide; DSC: Differential scanning calorimetry; FBS: Fetal bovine serum; IFIs: Systemic or invasive fungal infections; IONP: Iron oxide nanoparticle; MβCD: methyl-β-cyclodextrin; PBS: Phosphate buffer saline; TEM: Transmission electron microscope.

## References

[1] Hamill R.J. (2013). Amphotericin B formulations: A comparative review of efficacy and toxicity. Drugs.

[2] Moen M.D., Lyseng-Williamson K.A., Scott L.J. (2009). Liposomal amphotericin B: A review of its use as empirical therapy in febrile neutropenia and in the treatment of invasive fungal infections. Drugs.

[3] Kontoyiannis D.P., Marr K.A., Park B.J., Alexander B.D., Anaissie E.J., Walsh T.J., Ito J., Andes D.R., Baddley J.W., Brown J.M. (2010). Prospective surveillance for invasive fungal infections in hematopoietic stem cell transplant recipients, 2001–2006: Overview of the Transplant-Associated Infection Surveillance Network (TRANSNET) Database. Clin. Infect. Dis..

[4] Park B.J., Pappas P.G., Wannemuehler K.A., Alexander B.D., Anaissie E.J., Andes D.R., Baddley J.W., Brown J.M., Brumble L.M., Freifeld A.G. (2011). Invasive non-Aspergillus mold infections in transplant recipients, United States, 2001–2006. Emerg. Infect. Dis..

[5] Azie N., Neofytos D., Pfaller M., Meier-Kriesche H.U., Quan S.P., Horn D. (2012). The PATH (Prospective Antifungal Therapy) Alliance(R) registry and invasive fungal infections: Update 2012. Diagn. Microbiol. Infect. Dis..

[6] Park B.J., Chiller T.M., Brandt M.E., Warnock D.W. (2011). Epidemiology of systemic fungal diseases: An overview. Essentials of Clinical Mycology.

[7] Lewis R.E. (2011). Current concepts in antifungal pharmacology. Mayo Clinic Proceedings.

[8] Gupta A.K., Gupta M. (2005). Synthesis and surface engineering of iron oxide nanoparticles for biomedical applications. Biomaterials.

[9] Marcu A., Pop S., Dumitrache F., Mocanu M., Niculite C., Gherghiceanu M., Lungu C., Fleaca C., Ianchis R., Barbut A. (2013). Magnetic iron oxide nanoparticles as drug delivery system in breast cancer. Appl. Surf. Sci..

[10] Dilnawaz F., Singh A., Mohanty C., Sahoo S.K. (2010). Dual drug loaded superparamagnetic iron oxide nanoparticles for targeted cancer therapy. Biomaterials.

[11] Chen S. (2010). Polymer-Coated Iron Oxide Nanoparticles for Medical Imaging. Ph.D. Thesis.

[12] Balabathula P. (2015). Development and Evaluation of Amphotericin B Loaded Iron Oxide Nanoparticles for Targeted Drug Delivery to Systemic Fungal Infections. Ph.D. Thesis.

[13] U.S. Department of Health and Human Services Antibiotic Resistance Threats in the United States. http://www.cdc.gov/drugresistance/pdf/ar-threats-2013-508.pdf.

[14] Ocean NanoTech Carboxyl Magnetic Iron Oxide Nanoparticles Conjugation Kits. http://www.oceannanotech.com/upload/120924114444478894tegv6h.pdf.

[15] Ocean NanoTech Gel Electrophoresis Protocol. http://www.oceannanotech.com/upload/090604132955928413aza6sm.pdf.

[16] Zhang L., Chan J.M., Gu F.X., Rhee J.W., Wang A.Z., Radovic-Moreno A.F., Alexis F., Langer R., Farokhzad O.C. (2008). Self-assembled lipid--polymer hybrid nanoparticles: A robust drug delivery platform. ACS Nano.

[17] Balabathula P., Janagam D., Mittal N., Mandal B., Thoma L., Wood G. (2013). Rapid quantitative evaluation of amphotericin B in human plasma, by validated HPLC method. J. Bioequiv. Availab..

[18] Hu C.M.J., Zhang L., Aryal S., Cheung C., Fang R.H. (2011). Erythrocyte membrane-camouflaged polymeric nanoparticles as a biomimetic delivery platform. Proc. Natl. Acad. Sci. USA.

[19] Fang R.H., Aryal S., Hu C.M., Zhang L. (2010). Quick synthesis of lipid-polymer hybrid nanoparticles with low polydispersity using a single-step sonication method. Langmuir.

[20] Costa E.C., Gaspar V.M., Marques J.G., Coutinho P., Correia I.J. (2013). Evaluation of nanoparticle uptake in co-culture cancer models. PLoS ONE.

[21] Ibuki Y., Toyooka T. (2012). Nanoparticle uptake measured by flow cytometry. Methods Mol. Biol. (Cliftonn. J.).

[22] CLSI (2008). Reference Method for Broth Dilution Antifungal Susceptibility Testing of Yeasts. Approved Standard M27-A3.

[23] Kudva A.K., Manoj M., Swamy B., Ramadoss C. (2011). Complexation of amphoterecin B and curcumin with serum albumin: Solubility and effect on erythrocyte membrane damage. J. Exp. Pharmacol..

[24] Oliveira S., Schiffelers R.M., van der Veeken J., van der Meel R., Vongpromek R., van Bergen En Henegouwen P.M., Storm G., Roovers R.C. (2010). Downregulation of EGFR by a novel multivalent nanobody-liposome platform. J. Control. Release.

[25] Vacha R., Martinez-Veracoechea F.J., Frenkel D. (2011). Receptor-mediated endocytosis of nanoparticles of various shapes. Nano Lett..

[26] dos Santos T., Varela J., Lynch I., Salvati A., Dawson K.A. (2011). Effects of transport inhibitors on the cellular uptake of carboxylated polystyrene nanoparticles in different cell lines. PLoS ONE.

[27] Diaz-Moscoso A., Vercauteren D., Rejman J., Benito J.M., Ortiz Mellet C., De Smedt S.C., Fernandez J.M. (2010). Insights in cellular uptake mechanisms of pDNA-polycationic amphiphilic cyclodextrin nanoparticles (CDplexes). J. Control. Release.

[28] Rodal S.K., Skretting G., Garred O., Vilhardt F., van Deurs B., Sandvig K. (1999). Extraction of cholesterol with methyl-beta-cyclodextrin perturbs formation of clathrin-coated endocytic vesicles. Mol. Biol. Cell.

[29] Zhang L.W., Monteiro-Riviere N.A. (2009). Mechanisms of quantum dot nanoparticle cellular uptake. Toxicol. Sci..

[30] Ishida K., Cipriano T.F., Rocha G.M., Weissmüller G., Gomes F., Miranda K., Rozental S. (2014). Silver nanoparticle production by the fungus Fusarium oxysporum: Nanoparticle characterisation and analysis of antifungal activity against pathogenic yeasts. Mem. Do Inst. Oswaldo Cruz.

[31] Wu L., Yu X., Feizpour A., Reinhard B.M. (2014). Nanoconjugation: A Materials Approach to Enhance Epidermal Growth Factor Induced Apoptosis. Biomater. Sci..

[32] Jiang M., Gan L., Zhu C., Dong Y., Liu J., Gan Y. (2012). Cationic core-shell liponanoparticles for ocular gene delivery. Biomaterials.

[33] Ito T., Sun L., Bevan M.A., Crooks R.M. (2004). Comparison of Nanoparticle Size and Electrophoretic Mobility Measurements Using a Carbon-Nanotube-Based Coulter Counter, Dynamic Light Scattering, Transmission Electron Microscopy, and Phase Analysis Light Scattering. Langmuir.

[34] Barzegar-Jalali M., Adibkia K., Valizadeh H., Shadbad M.R., Nokhodchi A., Omidi Y., Mohammadi G., Nezhadi S.H., Hasan M. (2008). Kinetic analysis of drug release from nanoparticles. J. Pharm. Pharm. Sci. Publ. Can. Soc. Pharm. Sci. Soc. Can. Des Sci. Pharm..

[35] Modi S., Anderson B.D. (2013). Determination of drug release kinetics from nanoparticles: Overcoming pitfalls of the dynamic dialysis method. Mol. Pharm..

[36] Costa P., Sousa Lobo J.M. (2001). Modeling and comparison of dissolution profiles. Eur. J. Pharm. Sci..

[37] Tong R., Hemmati H.D., Langer R., Kohane D.S. (2012). Photoswitchable Nanoparticles for Triggered Tissue Penetration and Drug Delivery. J. Am. Chem. Soc..

[38] Kamiński D.M. (2014). Recent progress in the study of the interactions of amphotericin B with cholesterol and ergosterol in lipid environments. Eur. Biophys. J..

[39] Divi M.K. (2007). Development and Evaluation of Brain Tumor Targeted Liposome Delivery System for Paclitaxel. Ph.D. Thesis.

[40] Treuel L., Jiang X., Nienhaus G.U. (2013). New views on cellular uptake and trafficking of manufactured nanoparticles. J. R. Soc. Interface R. Soc..

[41] Kirkham M., Parton R.G. (2005). Clathrin-independent endocytosis: New insights into caveolae and non-caveolar lipid raft carriers. Biochim. Et Biophys. Acta (Bba) Mol. Cell Res..

[42] Xu X., Bittman R., Duportail G., Heissler D., Vilcheze C., London E. (2001). Effect of the structure of natural sterols and sphingolipids on the formation of ordered sphingolipid/sterol domains (rafts) Comparison of cholesterol to plant, fungal, and disease-associated sterols and comparison of sphingomyelin, cerebrosides, and ceramide. J. Biol. Chem..

[43] Doherty G.J., McMahon H.T. (2009). Mechanisms of endocytosis. Annu. Rev. Biochem..

[44] Sieczkarski S.B., Whittaker G.R. (2002). Dissecting virus entry via endocytosis. J. Gen. Virol..

[45] Thomsen P., Roepstorff K., Stahlhut M., van Deurs B. (2002). Caveolae are highly immobile plasma membrane microdomains, which are not involved in constitutive endocytic trafficking. Mol. Biol. Cell.

[46] Rejman J., Bragonzi A., Conese M. (2005). Role of clathrin- and caveolae-mediated endocytosis in gene transfer mediated by lipo- and polyplexes. Mol. Ther. J. Am. Soc. Gene Ther..

[47] McMahon H.T., Boucrot E. (2011). Molecular mechanism and physiological functions of clathrin-mediated endocytosis. Nat. Rev. Mol. Cell Biol..

[48] Choi C.H.J., Hao L., Narayan S.P., Auyeung E., Mirkin C.A. (2013). Mechanism for the endocytosis of spherical nucleic acid nanoparticle conjugates. Proc. Natl. Acad. Sci. USA.

[49] Gomes P.N., da Silva W.J., Pousa C.C., Narvaes E.A.O., Cury A.A.D.B. (2011). Bioactivity and cellular structure of *Candida albicans* and *Candida glabrata* biofilms grown in the presence of fluconazole. Arch. Oral Biol..

[50] Kannan V. (2010). Development and Evaluation of Paclitaxel-Loaded Liposomal Formulations for Targeted Drug Delivery to Breast Cancer. Ph.D. Thesis.

[51] Kannan V., Balabathula P., Thoma L.A., Wood G.C. (2014). Effect of sucrose as a lyoprotectant on the integrity of paclitaxel-loaded liposomes during lyophilization. J. Liposome Res..

[52] Abdelwahed W., Degobert G., Stainmesse S., Fessi H. (2006). Freeze-drying of nanoparticles: Formulation, process and storage considerations. Adv. Drug Deliv. Rev..

[53] Koudelka Š., Turánek-Knötigová P., MaŠek J., Korvasová Z., Škrabalová M., Plocková J., Bartheldyová E., Turánek J. (2010). Liposomes with high encapsulation capacity for paclitaxel: Preparation, characterisation and in vivo anticancer effect. J. Pharm. Sci..

[54] Loefgreen C., Stading M. (1997). Glass Transitions in frozen sucrose solutions. Annu. Trans. Nord. Rheol. Soc..

[55] Fuller B.J. (2004). Cryoprotectants: The essential antifreezes to protect life in the frozen state. Cryo Lett..

[56] Patro S.Y. (2004). Freeze-drying process development for protein pharmaceuticals. Lyophilization Biopharm..

[57] Breen E., Curley J., Overcashier D., Hsu C., Shire S. (2001). Effect of moisture on the stability of a lyophilized humanized monoclonal antibody formulation. Pharm. Res..

[58] May J.C., Wheeler R.M., Etz N., Del Grosso A. (1992). Measurement of final container residual moisture in freeze-dried biological products. Dev. Biol. Stand..

[59] FDA, CBER (1990). Guideline for the Determination of Residual Moisture in Dried Biological Products.

[60] Manosroi A., Kongkaneramit L., Manosroi J. (2004). Stability and transdermal absorption of topical amphotericin B liposome formulations. Int. J. Pharm..

