# Supplementary material for: Lyophilized Iron Oxide Nanoparticles Encapsulated in Amphotericin B: A Novel Targeted Nano Drug Delivery System for the Treatment of Systemic Fungal Infections
Source: Pharmaceutics. 2020 Mar 10;12(3):247. doi: 10.3390/pharmaceutics12030247 (PMC7150906; doi:10.3390/pharmaceutics12030247)
Supplement: Supplementary file 1 [file pharmaceutics-12-00247-s001.pdf]

pharmaceutics

Supplementary Materials: Lyophilized Iron Oxide Nanoparticles encapsulated Amphotericin B: A Novel Targeted Nano Drug Delivery System for the Treatment of Systemic Fungal Infections

Methods

S1. Formulation Preparation (Refereed in Section 2.2 of Main Article)

Surface activation of IONP: The oleic acid-coated IONP (1 mg) were initially treated with 100 µL of activation buffer, and then immediately the surface groups on IONP were activated with 100 µL of 2 mg/mL EDAC and 1 mg/mL sulfo-NHS. The mixture was reacted for 10 min with continuous mixing at ambient temperature. Then 400 µL of the coupling buffer was added to the activated IONP and mixed well.

Washing and storage of IONP-containing reaction mixtures: The reaction mixture was transferred into a plastic cuvette, 3 mL of wash/storage buffer added, and gently mixed well with a pipette. The cuvette was placed into the magnetic separator and the IONP was allowed to separate at 4 °C for 10-12 h. Without touching the inner walls of the cuvette, the liquid was carefully removed with a Pasteur pipette. In this way most of the free material unattached to the IONP removed. The cuvette was removed and the IONP resuspended in 3 mL of wash/storage buffer with gentle mixing with a pipette (second washing). Then the mixture was subjected to magnetic separation of the nanoparticles for another 10–12 h at 4 °C. The liquid was carefully aspirated and the BSA-coated IONP suspended in the desired solution or 400–500 µL of wash/storage buffer for storing at 4 °C until further usage.

Design A: The order of the layers from inside to outside are IONP, BSA, and AMB (Figure 1 design A in main article). At least 1 mg of BSA dissolved in a maximum volume of 0.5 mL was added to the activated IONP mixture, and the mixture was reacted for two hours with continuous mixing at ambient temperature. Then 10 µL of the quenching buffer was added, mixed well and incubated at ambient temperature for at least 10 minutes. AMB Coating: The BSA coated IONP were suspended in 0.5 mL AMB solution in DMSO (50–100 µg/mL). The mixture was incubated at ambient temperature for about 2 h for adsorption/binding of the drug to albumin to obtain AMB-IONP of design A. The product was then washed as described in the washing step procedure above and stored.

Design B: The order of the layers from inside to outside are IONP, AMB, and BSA (Figure 1 design B in main article). 0.5 mL of the AMB solution in DMSO (50–100 µg/mL) was added to the activated IONP mixture, and allowed to react for two hours with continuous mixing at ambient temperature. Then 10 µL of the quenching buffer was added, mixed well and incubated at ambient temperature for at least 10 minutes. Then the nanoparticles were washed as described in design A. Next, the AMB-coated IONP was suspended in 0.5 mL BSA solution (2–3mg/mL) in coupling buffer. The mixture was incubated at ambient temperature for 2 hours for binding of albumin to drug-coated IONP to obtain AMB-IONP of design B. Then the nanoparticles were washed as per procedure above and stored.

Design C: The order of the layers from inside to outside are IONP, AMB, BSA, and AMB (Figure 1 design C in main article). Design C is the extension of design B. So, after the preparation of design B, the nanoparticles are suspended in 0.5 mL AMB solution in DMSO (50–100 µg/mL) and incubated at ambient temperature for about two hours for binding of AMB to albumin on nanoparticles to obtain AMB-IONP of design C. Then the nanoparticles were washed as per procedure above and stored.

Design D: The order of the layers from inside to outside are IONP, AMB bound BSA (Figure 1 design D in main article). To ~0.5 mL of BSA solution (2–3mg/mL) in coupling buffer was added 40 µL of AMB (5 µg/mL), mixed gently and incubated for 1–2 h at ambient temperature to obtain drug-

bound albumin solution. Immediately, this 0.5 mL of AMB-bound BSA mixture was added to the surface-activated IONP mixture. The mixture was reacted for two hours with continuous mixing at ambient temperature. Then 10 μL of the quenching buffer was added, mixed well and incubated at ambient temperature for at least 10 minutes. Then the nanoparticles were washed as per procedure above and stored.

Design E: The order of the layers from inside to outside are IONP and AMB (Figure 1 design E main article). 0.5 mL of AMB solution (50–100 μg/mL) was added to the activated IONP mixture. The mixture was reacted for two hours with continuous mixing at ambient temperature. Then 10 μL of the quenching buffer was added, mixed well and incubated at ambient temperature for at least 10 minutes. Then the nanoparticles were washed as per procedure above and stored.

S2. Drug loading (Refereed in Section 2.2 of Main Article)

Loaded AMB amounts in the AMB-IONP were determined by a validated HPLC method developed previously [1]. All chromatographic studies were conducted on validated and qualified equipment. The HPLC system consists of a Waters HPLC Alliance system on an e2695 separations module with a Waters 2998 PDA detector (Milford, MA, USA). Samples were injected using a Waters auto-injector and the instrument was controlled by use of Empower2® software (Milford, MA, USA). Components were separated on a Waters XBridgeTM C18 reversed-phase column (Milford, MA, USA) with 150 mm × 4.6 mm dimensions and 3.5 μm particle size. The column was kept thermostatic at 30°C in a Waters column oven (Milford, MA, USA). The detection and quantification of AMB was performed using an isocratic method with a mobile phase of acetic acid (0.73%)-acetonitrile (59:41, v/v). The flow rate of the solvent was 1.0 mL/min with a runtime of 6 minutes. The drug was extracted from the formulation by dispersing the AMB-IONP in DMSO at a dilution factor of 25 and then subjecting to sonication for 30 minutes. The dispersion was then subjected to magnetic separation for 10–12 h at 4 °C. An aliquot was taken for HPLC analysis. Samples were injected into the column at a constant volume of 20 μL and a PDA detector at 408 nm was employed to obtain the response for AMB.

The AMB peak is well resolved from the adjacent peaks with a retention time of 2.7 min. The drug loaded into the formulations was directly determined from each formulation by extracting the drug into DMSO. The drug loading was determined by amount of drug in nanoparticles over amount of nanoparticles. The amount of drug loaded in the formulations was calculated from a standard curve which was linear between 0.050 to 1 μg/mL with a coefficient of determination (r^2) value of 0.9987.

S3. Particle size and Zeta potential (Refereed in Section 2.2 of Main Article)

The mean hydrodynamic size (Z-average) of IONP and AMB-IONP were determined by a dynamic light scattering (also known as photon correlation spectroscopy) technique using Malvern Zetasizer Nano ZS90 (Westborough, MA, USA) equipped with 50 mW diode laser as the light source, operating at 532/633 nm. Particle-scattered photons were detected at an angle of 90°. Each sample was determined in duplicate. ζ-potential was estimated with an electrophoretic light scattering (also known as laser Doppler microelectrophoresis) technique with the Malvern Zetasizer Nano ZS90.

S4. In Vitro Drug Release Technique (Refereed in Section 2.5 of Main Article)

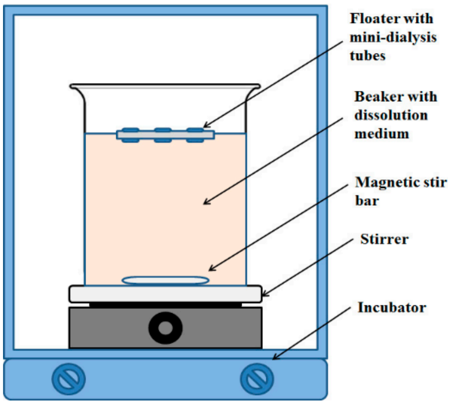

Figure S1. Schematic diagram of the in-house experimental setup for *in vitro* drug release study.

S5. Physicochemical Characterization of Lyophilized AMB-IONP (Refereed in Section 2.16 of Main Article)

The lyophilized product of the AMB-IONP was subjected to visual inspection to determine height of the cake, any cake separation, and product on rubber closures, meltback, and presence of any surface abnormalities such as air bubbles or puffing.

The lyophilized cake was then subjected to moisture content analysis using a Mettler Toledo DL31 Karl Fischer Titrator (Mettler Toledo Inc., Boston, MA, USA). The equipment was calibrated before use on the day of analysis. The sample of about 5 mg of the lyophilized product was weighed accurately and added to the titration mixture. The measurements were performed in triplicate and the moisture content in the lyophilized cake was calculated as % *w/w*.

The lyophilized product was reconstituted with water and the time for re-dispersibility after reconstitution was recorded. The mean hydrodynamic size (Z-average) and ζ-potential of the reconstituted AMB-IONP were determined.

Results and Discussion

S6. Drug Release Proile (Refereed in Section 3.4 of Main Article)

Table S1. In vitro release kinetics parameters of AMB from the IONP formulations.

Formulation	Zero Order		First Order		Higuchi		Hixson-Crowell		Korsmeyer-Peppas	
	r^2	k	r^2	k	r^2	k	r^2	k	r^2	N
Design A	0.4103	0.7455	0.9545	0.0094	0.9176	4.1335	0.7884	0.0069	0.9663	0.1207
Design B	0.6024	0.9222	0.822	0.0094	0.7904	7.6315	0.5579	0.015	0.8058	0.3317
Design C	0.8492	1.1405	0.9953	0.0134	0.9652	10.702	0.8111	0.0212	0.9572	0.5172
Design D	0.7922	0.8244	0.9629	0.0068	0.9659	6.972	0.744	0.0203	0.9583	0.2729
Design D+G	0.8817	0.7956	0.9605	0.0054	0.9694	7.0278	0.6495	0.029	0.8597	0.5776

S5. Schematic Digram of Different Cellular Uptake Mechanisms (Refereed in Section 3.9 of Main Article)

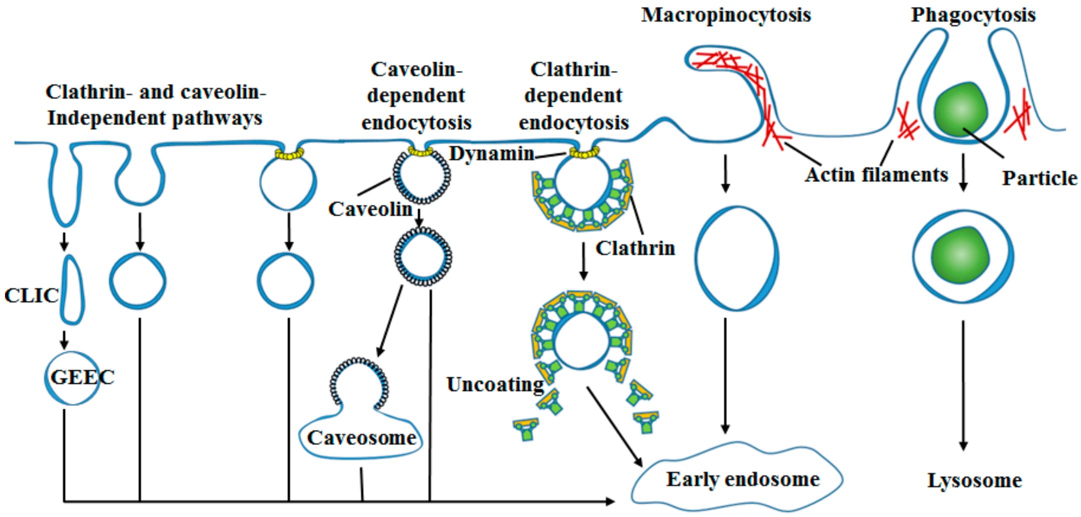

Figure S2. Schematic representation of different cellular uptake pathways. (Notes: Uptake of large size particles usually occurs by phagocytosis, while fluid is taken up by the macropinocytosis pathway. Both pathways are commonly initiated by and are reliant on actin-mediated transformation of the plasma membrane at a large scale. The vesicle sizes formed by these processes are much larger when compared to the vesicles formed by other endocytic pathways. Clathrin-mediated endocytosis is a receptor-mediated endocytosis process through the formation of clathrin-coated pits. Caveolae-mediated endocytosis is also a receptor-mediated endocytosis process with formation of invaginated, vesicular/tubulovesicular or flask-shaped vesicles, known as caveolae. Clathrin- and caveolin-independent endocytosis have vesicle formation of about 50 nm. These pathways require specific lipid compositions, such as cholesterol or ergosterol (in case of yeast cells). Various cargoes can be endocytosed by pathways, which are independent of the clathrin (coat protein) and dynamin (fission GTPase). Most of the internalized cargoes are transported to the early endosome through clathrin- or caveolin-coated vesicles (vesicular) or clathrin- and dynamin-independent carriers (CLICs) (tubular intermediates), which are derived from the plasma membrane. Before trafficking to the early endosome, some mechanisms may first traffic to intermediate compartments and include caveosome or glycosyl phosphatidylinositol-anchored protein enriched early endosomal compartments (GEEC)).

S6. Intracellular Trafficking Localization of AMB-IONP in C. Albicans and C. Glabrata by Confocal Microscopy. (Refereed in Section 3.10 of Main Article)

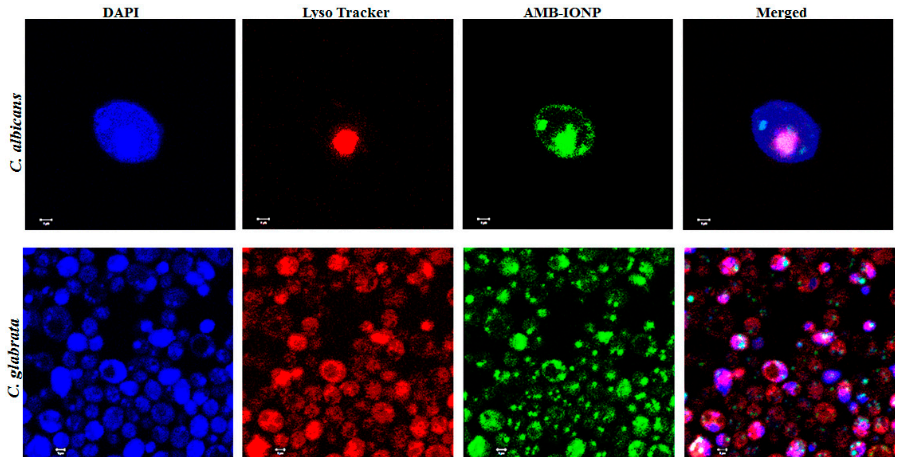

Figure S3. Intracellular trafficking localization of AMB-IONP in *C. albicans* and *C. glabrata* by confocal microscopy. (The confocal fluorescence revealed the colocalization of fluorescence signals from nucleus (blue), endosomes and lysosomes (deep red), and fluorescent labeled AMB-IONP (green), after 4 h of incubation of clinical isolates with nanoparticles.).

S6. DSC Thermograms of Different Sucrose Weight Ratios to IONP (Refereed in Section 3.11 of Main Article)

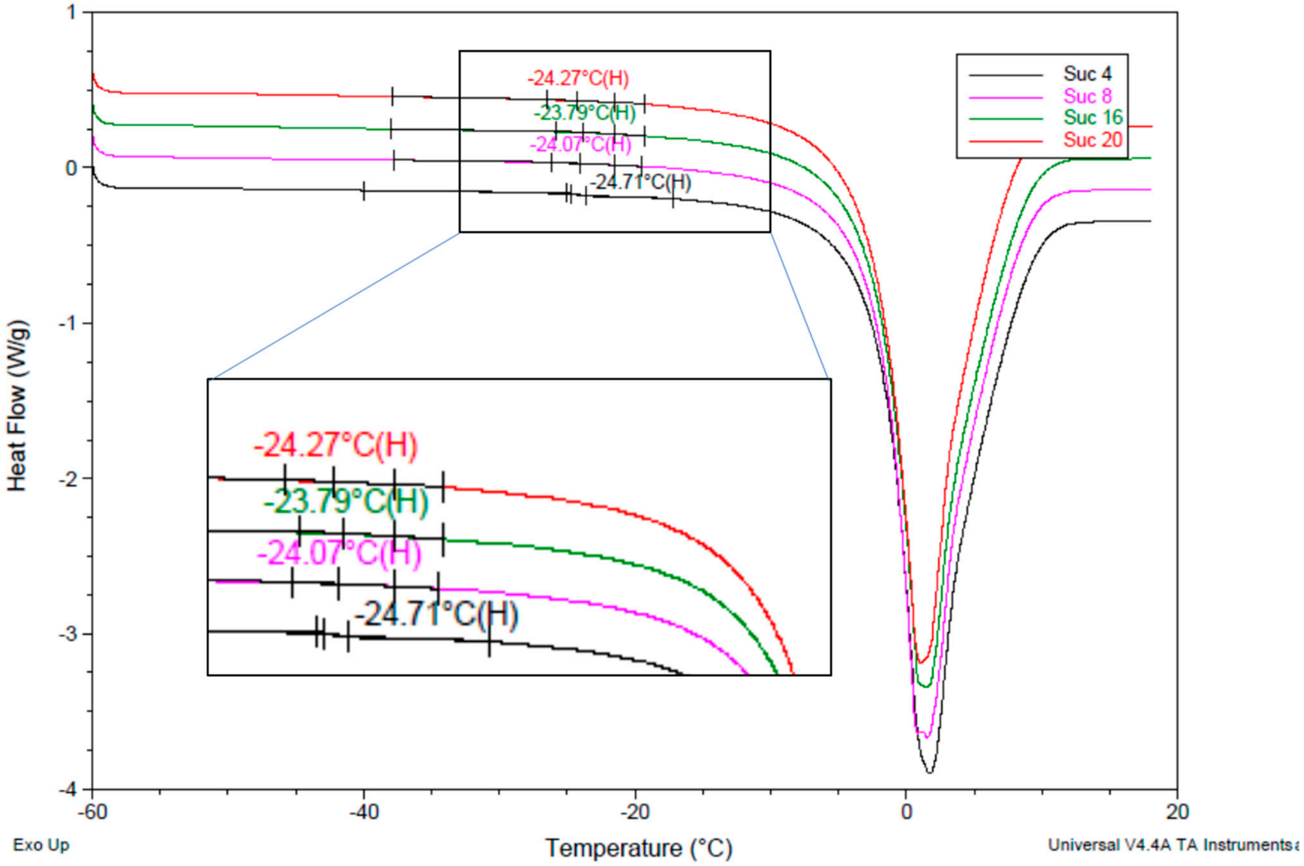

Figure S4. DSC thermograms of different sucrose weight ratios to IONP. (Notes: The data indicates that the Tg' values increased with an increase of sucrose weight ratio in the mixture. The Tg' value was found to be about –25 °C. Thus, primary drying temperature of 2–5 °C < Tg' was used to inhibit collapse.).

S7. DSC th [illegible] *article)*

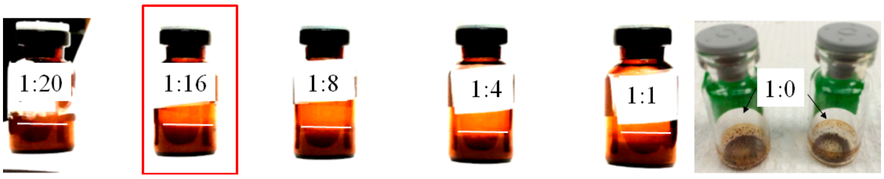

Figure S5. Physical appearance of lyophilized product of AMB-IONP with different weight ratios of sucrose. (Sucrose weight ratio of 16 and higher found to be optimum without any visible abnormalities of the cake).

References

1. Balabathula, P.; Janagam, D.; Mittal, N.; Mandal, B.; Thoma, L.; Wood, G. Rapid quantitative evaluation of amphotericin B in human plasma, by validated HPLC method. *J Bioequiv Availab* **2013**, *5*, 121-124.

© 2020 by the authors. Submitted for possible open access publication under the terms and conditions of the Creative Commons Attribution (CC BY) license (http://creativecommons.org/licenses/by/4.0/).
